# Effective range of non-cell autonomous activator and inhibitor peptides specifying plant stomatal patterning

**DOI:** 10.1242/dev.192237

**Published:** 2020-09-11

**Authors:** Scott M. Zeng, Emily K. W. Lo, Bryna J. Hazelton, Miguel F. Morales, Keiko U. Torii

**Affiliations:** 1 Department of Physics, University of Washington, Seattle, WA 98195, USA; 2 Department of Biology, University of Washington, Seattle, WA 98195, USA; 3 eScience Institute, University of Washington, Seattle, WA 98195 USA; 4 Howard Hughes Medical Institute, University of Texas at Austin, Austin, TX 78712, USA; 5 Department of Molecular Biosciences, University of Texas at Austin, Austin, TX 78712, USA

**Keywords:** *Arabidopsis*, Genetic mosaic, Mathematical analysis, Peptide signaling, Spacial autocorrelation, Stomata

## Abstract

Stomata are epidermal valves that facilitate gas exchange between plants and their environment. Stomatal patterning is regulated by the EPIDERMAL PATTERING FACTOR (EPF) family of secreted peptides: EPF1 enforces stomatal spacing, whereas EPIDERMAL PATTERNING FACTOR-LIKE9 (EPFL9), also known as Stomagen, promotes stomatal development. It remains unknown, however, how far these signaling peptides act. Utilizing Cre-lox recombination-based mosaic sectors that overexpress either EPF1 or Stomagen in *Arabidopsis* cotyledons, we reveal a range within the epidermis and across the cell layers in which these peptides influence patterns. To determine their effective ranges quantitatively, we developed a computational pipeline, SPACE (stomata patterning autocorrelation on epidermis), that describes probabilistic two-dimensional stomatal distributions based upon spatial autocorrelation statistics used in astrophysics. The SPACE analysis shows that, whereas both peptides act locally, the inhibitor EPF1 exerts longer range effects than the activator Stomagen. Furthermore, local perturbation of stomatal development has little influence on global two-dimensional stomatal patterning. Our findings conclusively demonstrate the nature and extent of EPF peptides as non-cell autonomous local signals and provide a means for quantitative characterization of complex spatial patterns in development.

This article has an associated ‘The people behind the papers’ interview.

## Abstract (precis)

**Highlighted Article:** Non-cell autonomous effects of activator and inhibitor peptides on 2D spatial patterning of stomata were quantitatively characterized using chimeric sectors and a SPACE computational pipeline.

## INTRODUCTION

During the development of multicellular organisms, distinct cell types emerge with specific roles and functions. Cell-to-cell communication of positional cues and spatial information is essential for coordinating the transition from a tissue of uniformly undifferentiated cells into a robust pattern of cells with specialized identities. For plant systems, the cell wall prevents direct cell-to-cell contact or cell mobility, thereby excluding many of the mechanisms for pattern formation studied in animals, such as the transmembrane receptor Notch and its membrane-bound ligand Delta (Artavanis-Tsakonas et al., 1999), or the contact-dependent depolarization and repulsion between different pigment cell types in zebrafish stripe patterning (Eom and Parichy, 2017; Inaba et al., 2012; Nusslein-Volhard, 2012). The absence of these mechanisms means that plants are model systems for isolating and studying the role of local ligand secretion in pattern formation independently of variables such as cell movement or apoptosis (Torii, 2012b).

Stomata, the pores on the plant epidermis responsible for mediating gas exchange and water control, differentiate according to a special cue, which enforces the ‘one-cell spacing rule’ in which no two stomata develop adjacent to one other (Bergmann and Sack, 2007; Pillitteri and Torii, 2012). Locally, stomatal spatial patterning is enforced through a family of small secreted peptide ligands called EPIDERMAL PATTERNING FACTORS (EPFs), which are perceived by a family of ERECTA receptor kinases and their signal modulator TOO MANY MOUTHS (TMM) (Hara et al., 2007, 2009; Hunt and Gray, 2009; Lee et al., 2012; Nadeau and Sack, 2002; Shpak et al., 2005; Torii, 2012a). Perception of EPF2 peptide inhibits the entry into stomatal cell lineages (Hara et al., 2009; Hunt and Gray, 2009). Antagonistically, EPIDERMAL PATTERNING FACTOR-LIKE9 (EPFL9), also known as Stomagen, is secreted from the subepidermal tissue into the epidermis and promotes stomatal differentiation via competition for receptor binding with EPF2, and probably also with EPF1 (Kondo et al., 2010; Lee et al., 2015; Sugano et al., 2010). At a later stage, spatial patterning of stomata and differentiation of a stomatal precursor, known as a meristemoid, is controlled by EPF1 (Hara et al., 2007; Qi et al., 2017). Consistent with their function as signaling ligands controlling stomatal development, ectopic overexpression or peptide application of EPF1 or EPF2 results in epidermis devoid of stomata, the former with arrested meristemoids and the latter with reduced stomatal lineage cells (Hara et al., 2007, 2009; Hunt and Gray, 2009). Conversely, *STOMAGEN* overexpression or peptide application produces stomatal clusters, resembling the loss of *TMM* or three *ERECTA*-family genes (Kondo et al., 2010; Sugano et al., 2010).

Globally, long range signals are also necessary to optimize stomatal patterning for its physiological functions of mediating gas exchange, water exchange and photosynthetic efficiency (Hetherington and Woodward, 2003). Small chemical hormones such as ethylene increase stomata (Serna and Fenoll, 1996) whereas others such as abscisic acid reduce their number (Tanaka et al., 2013), but the effect of these individual chemicals can depend on the tissue or species (Qi and Torii, 2018). Auxin is another hormone that broadly regulates plant development, but its inhibition of stomatal density partly depends on the absence of light, illustrating the integration of environmental information as another set of signals (Balcerowicz et al., 2014; Hronkova et al., 2015; Zhang et al., 2014). Furthermore, environmental factors perceived in mature leaves may affect stomatal density in younger leaves, demonstrating a spatial propagation of signaling that connects local and global contexts of patterning (Casson and Gray, 2008).

Whereas endogenous and environmental factors controlling stomatal development have been described in detail, much less well understood is how these signals propagate their efficacy in cell-to-cell communication to constitute the emergence of stomatal spatial patterning across the epidermis. The expression of *STOMAGEN* in the mesophyll indicates non-cell-autonomous effects across tissue layers, but the range of these signals as they travel between cells is unknown. One way to assess the movement of signaling peptides is to visualize their movement directly. However, the addition of fluorescent protein tags, such as GFP, impairs the movement of peptide hormones, and the highly processed nature of some peptides hampers such an approach. Moreover, such visualization does not address how and to what extent signaling peptides influence the local spatial patterning of stomata or whether there is any intersection with global epidermal patterning.

To address these questions, we harnessed Cre-lox recombination and the GAL4/UAS transactivation system (Heidstra et al., 2004) to generate mosaics in which peptide overexpression was localized to sectors of epidermal tissue. The idea here was to test how far EPF1 and Stomagen can influence stomatal patterning if they are expressed at equivalent levels driven by the same promoter in similarly confined areas (i.e. mosaic sectors). To analyze these effective ranges quantitatively, we developed SPACE (stomata patterning autocorrelation on epidermis), a computational pipeline that applies spatial correlation techniques. In contrast to traditional stomatal phenotype metrics such as stomata index or density, neither of which describes two-dimensional (2D) spatial patterning, our SPACE analysis revealed the effective range of EPF and Stomagen peptides in influencing epidermal patterning. Our study establishes the roles of EPF-family peptides as signals for cell-to-cell communication and the ranges at which they act. Our study also highlights the use of a spatial correlation approach for analysis of stomata patterning that can be adapted for analysis of both local and global signals, addressing the growing need for such techniques in phenotypic analysis of pattern formation.

## RESULTS

### Genetic mosaic analysis demonstrates non-cell-autonomous actions of EPF1 and STOMAGEN

**Fig. 1.**
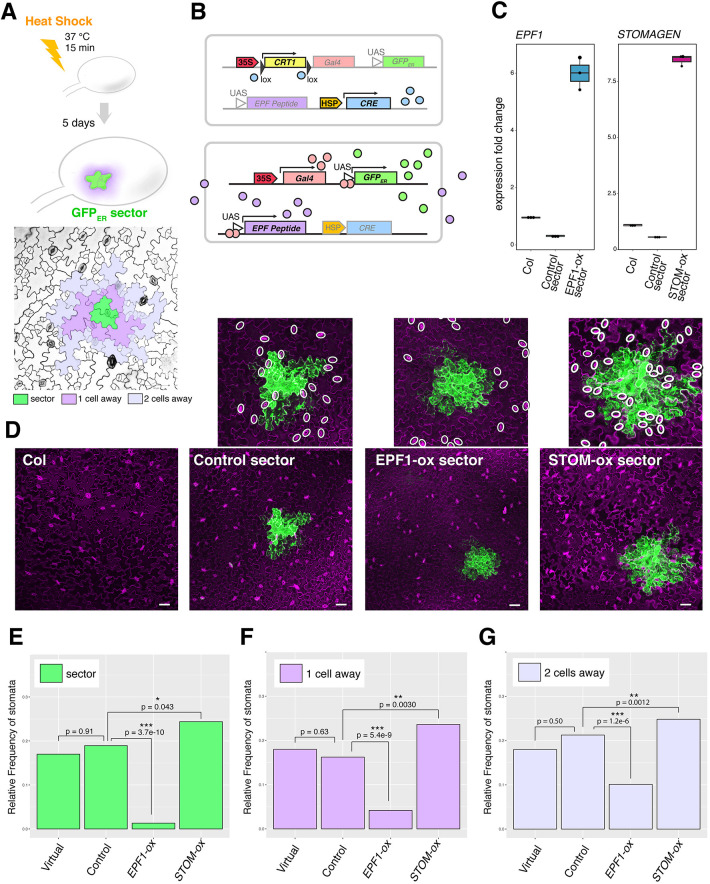
**Mosaic sectors overexpressing EPF peptides non-cell autonomously influence stomatal patterning.** (A) Top: Experimental design for generating ER-trapped GFP (GFP_ER_) sectors by heat-shock treatment. Bottom: False-colored confocal microscopy image of an abaxial epidermis; green, a sector; purple, cells immediately adjacent to the sector (one cell away); lilac, cells two cells away from the sector. (B) Scheme of heat-shock induced Cre-lox recombination and induction of GFP_ER_ as well as secreted EPF peptides. Top: Heat-shock promoter drives expression of CRE recombinase (light blue), which acts on lox recombination sites (black triangles) to cleave off the *E.uredovora CRT1* gene (yellow). Bottom: Removal of insulator gene *CRT1* now drives *Gal4* (peach) under the control of CaMV35S promoter. The Gal4 protein binds to the UAS at the promoter of GFP_ER_ (green) as well as *EPF* peptide gene (purple). The resulting GFP_ER_ protein marks the mosaic sectors, whereas EPF peptides are secreted to the apoplast. (C) Quantitative RT-PCR analysis of transcripts of *EPF1* (left) and *STOMAGEN* (right) from 7-day-old seedlings of non-transformed Col, control sector expressing GFP_ER_ only and sectors expressing *EPF1* (EPF1-ox) or *STOMAGEN* (STOM-ox). The transcripts are normalized against Actin expression (*ACT2*). Three biological replicates were performed, each with three technical replicates, and representative results are shown. (D) Z-stacked, tile-scanned representative confocal microscopy images of 7-day-old cotyledons subjected to heat-shock treatment as described in A. From left, non-transformed Col-0, transgenic lines expressing a control sector, EPF1-ox sector, and STOM-ox sector. Insets above show close-up images of each sector with stomata highlighted by white ovals. For tile scan of the entire cotyledons, see Fig. S1. (E-G) Relative frequency of stomata (number of stomata per total number of epidermal cells) within sectors (E; green), cells immediately adjacent to sectors (F; purple, ‘one cell away’), and cells adjacent to immediate neighboring cells (G; lilac, ‘two cells away’). For wild type cotyledons, virtual geometric sectors of the same size and geometry as real sectors were computationally placed. Total numbers of stomata and epidermal cells were aggregated to generate a single dataset for each genotype to enable robust statistical testing. Numbers of sectors subjected to analysis: *n*=20 (geometric), *n*=34 (control), *n*=25 (EPF1-ox), *n*=31 (STOM-ox). Total numbers of stomata and non-stomatal epidermal cells counted in sectors: *n*=229 (geometric), *n*=364 (control), *n*=228 (EPF1-ox), *n*=410 (STOM-ox). Total numbers of stomata and non-stomatal epidermal cells counted adjacent to sectors: *n*=384 (geometric), *n*=564 (control), *n*=358 (EPF1-ox), *n*=817 (STOM-ox). Total numbers of stomata and non-stomatal epidermal cells counted adjacent to immediate neighboring cells: *n*=702 (geometric), *n*=952 (control), *n*=633 (EPF1-ox), *n*=1398 (STOM-ox). A χ-square analysis was performed to test significant deviation between the frequencies of two aggregated samples;*P<0.05, **P<0.005, ***P<0.0005. Scale bars: 50 µm.

To address how EPF and EPFL peptides spatially influence stomatal patterning in a non-cell autonomous manner, we generated seedlings with genetic mosaic sectors overproducing individual EPF and EPFL peptides of opposite biological functions: EPF1, which restricts stomatal development, and Stomagen, which promotes stomatal development (Hara et al., 2007; Sugano et al., 2010). For this purpose, we implemented Cre-lox recombination coupled with the two-component GAL4/UAS transactivation system (Heidstra et al., 2004). Here, heat-shock treatment induces the expression of a CRE recombinase, which acts on two Lox-p sites to create GAL4+ sectors. Within the sectors, both endoplasmic reticulum-trapped green fluorescent protein (GFP_ER_), which marks the sectors in a cell autonomous manner, and EPF or EPFL peptide genes (either *EPF1* or *STOMAGEN/EPFL9*) were simultaneously overexpressed (Fig. 1A,B). For accurate monitoring of the non-cell autonomous effects of the EPF and EPFL genes, we expressed the non-epitope tagged EPF1 and STOMAGEN rather than a fluorescent protein fusion (e.g. CFP/RFP) that might impact the behavior of these small secreted peptides. Our heat-shock conditions yielded a high frequency of genetic mosaics per seedlings screened (13.9-100%; Table S1). Durations of heat-shock treatment were carefully analyzed to yield sectors of comparable size and number per cotyledon (see ‘Materials and Methods’). Quantitative reverse-transcription PCR (qRT-PCR) analysis confirmed that our heat-shock treatment led to elevated expression of *EPF1* and *STOMAGEN* transcripts (Fig. 1C).

To confirm that GFP_ER_ expression alone does not affect stomatal patterning or density, we also heat-shocked seedlings harboring control empty-vector to generate control GFP sectors that did not overexpress EPF1 or STOMAGEN (Fig. 1, Fig. S1). Furthermore, to determine whether the shape of sectors affects quantification, sector outlines from mosaics were overlaid onto heat shocked wild-type cotyledons to create virtual ‘geometric sectors’ as another control.

We first analyzed the stomatal phenotype within GFP_ER_-marked sectors (Fig. 1D-G). As expected, stomatal index (number of stomata/(number of stomata+non-stomatal epidermal cells) ×100) was significantly reduced within the EPF1-expressing sectors (*P*=3.7e-10) whereas it increased within the STOMAGEN-expressing sectors (*P*=0.043) (Fig. 1E). No statistical difference was observed between the stomatal index within control empty sectors and the geometric sectors overlaid onto the wild type (*P*=0.91) (Fig. 1E), confirming that heat shock treatment or GFP_ER_ expression does not influence stomatal development, and that sector shape does not bias quantification. Within the STOMAGEN sectors, stomata developed in clusters (Fig. 1D), thus verifying that our sector overexpression functioned as intended (Hara et al., 2007; Kondo et al., 2010; Lee et al., 2015, 2012; Sugano et al., 2010).

Next, we examined whether stomatal development in epidermal tissue proximal to the GFP_ER_ sectors was inhibited or promoted by peptide overexpression from these sectors. Confocal images of cotyledon epidermis showed that regions surrounding EPF1 sectors tended to be devoid of stomata, whereas regions surrounding STOMAGEN sectors differentiated more stomata (Fig. 1D). For quantitative analysis, we measured the stomatal index within each sector (Fig. 1A, green in diagram), adjacent to a sector (Fig. 1A, purple in diagram) and neighboring a sector-adjacent cell (Fig. 1A, lilac in diagram). Indeed, the stomatal index of cells adjacent to EPF1*-*expressing sectors was reduced (*P*=5.4e-9) and the stomatal index near to STOMAGEN*-*expressing sectors was increased (*P*=0.0030) (Fig. 1E). On the other hand, the stomatal index in cells adjacent (one cell away) to control empty sectors or neighboring the adjacent cells (two cells away) was not statistically different from either control empty sectors or heat-shocked wild-type geometric sectors (Fig. 1F,G). However, whereas the stomatal index near EPF1-expressing sectors remained lower than in control empty or wild-type geometric sectors, stomata production gradually increased farther away from EPF1 sectors (Fig. 1G), suggesting that the impact of a sector on stomatal patterning weakens with distance. Because each sector exhibits variable size and geometry, analysis of individual sectors without data aggregation increased the variations (Fig. S1D-F). Nevertheless, individual sector analysis also showed statistical significance for all EPF1-overexpressing sector data compared with that of control sectors, as well as that of Stomagen-overexpressing sectors compared with control sectors, except for the sectors one cell away (Fig. S1D-F). Combined, these mosaic sector analyses directly demonstrate the non-cell autonomous actions of EPF and EPFL peptides in adjacent and nearby epidermal cells.

### EPF1 secreted from the mesophyll can inhibit stomatal development

**Fig. 2.**
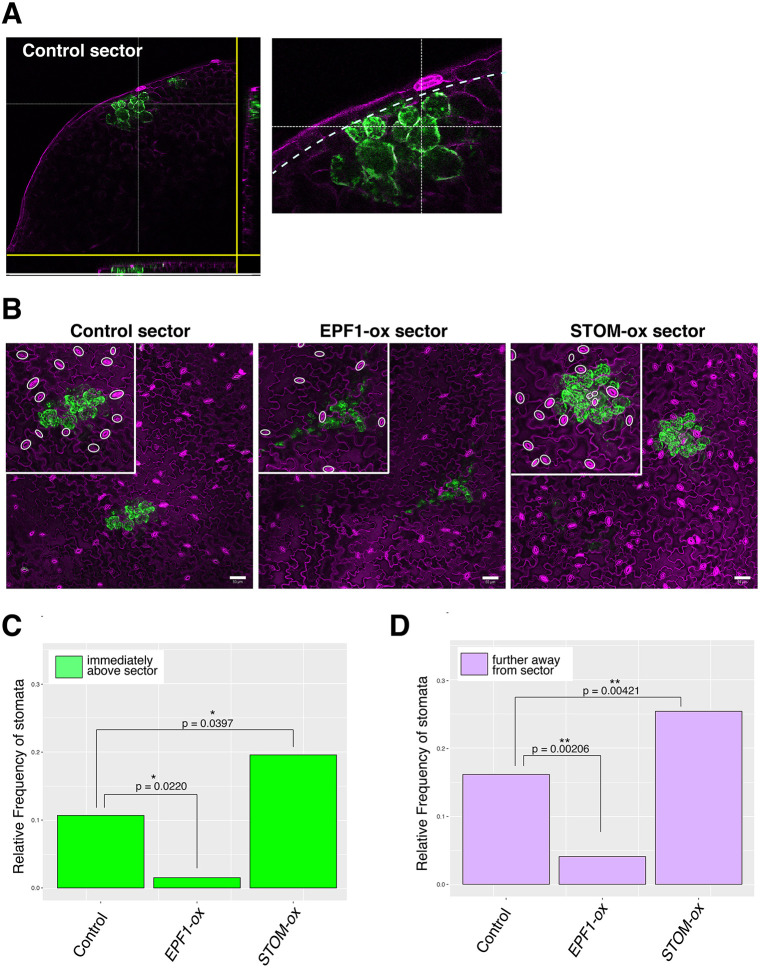
**Mesophyll sectors overexpressing EPF-family peptides locally influence stomatal patterning.** (A) Example of Cre-lox generated mesophyll sector shown as orthogonal slices. Right, close-up of a sector. Dotted line indicates an inner boundary of the epidermal layer. (B) Z-stacked, tile-scanned representative confocal microscopy images of 7-day-old cotyledons with mesophyll sectors. From left, transgenic lines expressing a control sector, EPF1-overexpressing sector (EPF1-ox) and Stomagen-overexpressing sector (STOM-ox). Insets above show close-up images of each sector with stomata highlighted by white ovals. For tile scan of the entire cotyledons, see Fig. S2. (C,D) Relative frequency of stomata (number of stomata per total number of epidermal cells) immediately above the mesophyll sector (C; green) and cells immediately adjacent to the cells above the mesophyll sectors (D; purple). Total numbers of stomata and epidermal cells were aggregated to generate a single dataset for each genotype to enable robust statistical testing. Number of sectors subjected to analysis: *n*=19 (control), *n*=8 (EPF1-ox), *n*=19 (STOM-ox). Total numbers of stomatal and non-stomatal epidermal cells subjected to analysis: *n*=295 (immediately above the mesophyll sector), *n*=773 (adjacent to cells immediately above the mesophyll sector). A χ-square analysis was performed to test significant deviation from the stomatal frequency of control plants; *P<0.05, **P<0.005. Scale bars: 50 µm.

Stomagen is known to secrete from the developing mesophyll layer to promote stomatal development in the epidermis (Kondo et al., 2010; Sugano et al., 2010). To address whether the EPF/EPFL-family peptides have an intrinsic property to function across tissue layers, we tested whether EPF1 expressed in the mesophyll could also affect stomatal development in the epidermis. For this purpose, we identified GFP_ER_ sectors induced exclusively in the mesophyll (Fig. 2A,B; Fig. S2A-C) and subsequently measured the stomatal index in the adaxial epidermal cells located directly above these sectors (Fig. 2C). As expected, Stomagen expression from mesophyll sectors promoted stomatal development; conversely, EPF1-expressing mesophyll sectors inhibited stomatal development in adjacent epidermal cells (Fig. 2C). As before, we extended our quantification to address whether this disruption to stomatal patterning acted at a larger range in cells that did not directly neighbor the mesophyll cells of interest (Fig. 2D). Likewise, although individual variations among mesophyll sector size and shape are large, unaggregated analysis of stomatal index still showed significant effects of these peptides (Fig. S2D,E). We conclude that, like Stomagen, EPF1 is capable of influencing the epidermis via secretion from the mesophyll in a non-cell-autonomous way if ectopically expressed.

### EPF1 and Stomagen act in a limited effective range

**Fig. 3.**
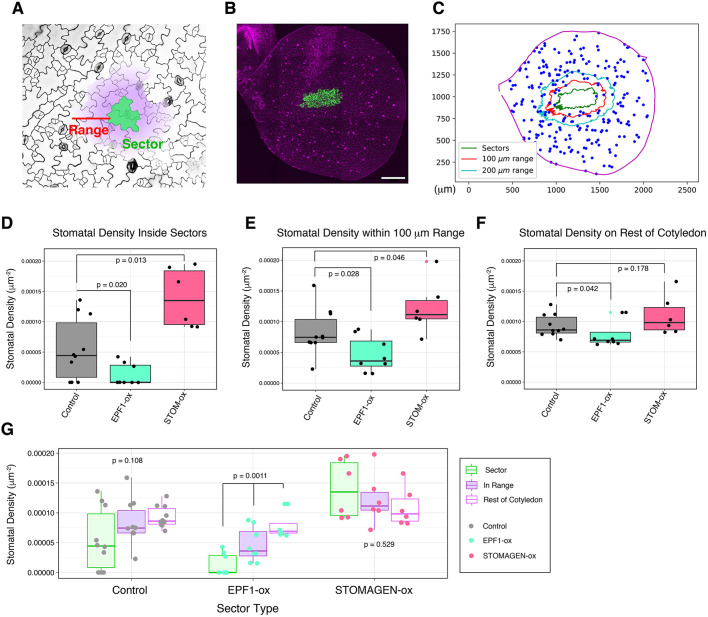
**Quantitative analysis of effective range by non-cell autonomous effects of EPF1-** **and Stomagen-overexpressing sectors.** (A) The coordinate outline of a given sector (green) was enlarged to generate a new coordinate boundary at a defined range (red; in this case, 100 µm) away from the sector outline. The defined range maintains the geometry of the original sector. The stomatal density was calculated in three regions: the interior of the original sector outline (green), the interior of the expanded range, excluding the original sector (purple, but not green); and the rest of the cotyledon (white). (B) Z-stacked, representative tile scan of a whole cotyledon with sectors overexpressing EPF1 (EPF1-ox). For tile scans of other genotypes, see Fig. S1. (C) Representative 2D coordinate mapping of the cotyledon boundary (magenta), the sector boundaries (green), the boundaries of expanded ranges at 100 µm (red) and 200 µm, and stomata (blue). Note that if the expanded range extends beyond the actual cotyledon boundaries, this extended area is excluded from analysis. (D) Stomatal density inside of sectors for individual cotyledons: cotyledons with control sector(s) expressing GFP_ER_ only (gray; *n*=10); cotyledons with EPF1-ox sector(s) (teal; *n*=8); cotyledons with sectors overexpressing Stomagen (STOM-ox) (coral red; *n*=6). Total numbers of stomata counted in sectors: *n*=24 (control), *n*=5 (EPF1-ox), *n*=50 (STOM-ox). A Mann–Whitney *U*-test was performed to test significant deviation between distributions of stomatal density. (E) Stomatal density within 100 µm range of sectors for individual cotyledons; cotyledons analyzed are same as in D. Total numbers of stomata counted within 100 µm range: *n*=116 (control), *n*=52 (EPF1-ox), *n*=148 (STOM-ox). A Mann–Whitney *U*-test was performed to test significant deviation between distributions of stomatal density. Red dot indicates an outlier (>Q3+1.5×IQR) for STOM-ox sector. (F) Stomatal density outside of the specified regions in D and E for individual cotyledons; cotyledons analyzed are the same as in D. Total numbers of stomata counted on the remaining area of cotyledons: *n*=2229 (control), *n*=1765 (EPF1-ox), *n*=1306 (STOM-ox). A Mann–Whitney *U*-test was performed to test significant deviation between distributions of stomatal density. Lime green dot indicates an outlier (>Q3+1.5×IQR) for EPF1-ox sector. (G) Data provided in D-F, grouped by sector type and region of stomatal density. For each sector type, a Kruskal–Wallis (non-parametric ANOVA) test was performed to test significant deviation in stomatal density in sectors versus 100 µm range versus rest of cotyledon. Scale bar: 250 µm.

Our results demonstrate that EPF1 and Stomagen act non-cell-autonomously, but do not address the distance at which these peptides can act to influence epidermal cell fate. We first analyzed this effective range by developing a computational pipeline to analyze the stomatal density quantitatively at various distances relative to the sectors (see ‘Materials and Methods’). Briefly, full tile-scanned confocal images of entire cotyledons were first converted into a 2D spatial coordinate plot of the XY-coordinates of every single stoma of an entire cotyledon, sector outlines and cotyledon outlines (Fig. 3A-C). Subsequently, stomatal density was calculated in the following regions: epidermal tissue inside the GFP_ER_ sector outline (‘the sector region’), epidermal tissue located within a 100 μm range of the GFP_ER_ sector outline excluding the sector interior itself (‘the nearby region’) and the remaining epidermal tissue beyond the 100 μm range (‘the faraway region’) (Fig. 3C). Because a given cell has a discrete size, we first selected the 100 μm cutoff to ascertain that the range was more than a given cell boundary. In our experimental system, the average size of stomata was 22.30±4.29 μm (longer side; *n*=60) and 15.32±2.22 μm (shorter side; *n*=60), the average size of non-stomatal epidermal cells was 87.11±30.92 μm (longer side; *n*=60) and 19.97±6.73 μm (shorter side; *n*=60) and the average one-cell spacing was 40.9±15.65 μm (*n*=60). Thus, on average, 100 μm corresponds to ∼2.45 cell-spacing, indicating that we are observing multicell range events.

As observed previously via stomatal index, the stomatal density inside EPF1 sectors was reduced and the stomatal density inside STOMAGEN sectors increased compared with the control, empty vector sectors (*P*=0.020 for EPF1 sectors and 0.013 for STOMAGEN sectors) (Fig. 3D). In the nearby region of a 100 μm range around sectors, the effect of these peptides on stomatal density remained statistically significant (*P*=0.028 for EPF1 sectors and *P*=0.046 for STOMAGEN sectors) (Fig. 3E), suggesting that non-cell autonomous actions of these secreted EPF and EPFL peptides are not constrained by cellular geometry. However, STOMAGEN sectors did not impact the stomatal density of the faraway region (beyond 100 μm) in a statistically significant manner (*P*=0.178) whereas EPF1 sectors did (*P*=0.042), relative to the stomatal density far away from control sectors (Fig. 3F). The results suggest that the non-cell-autonomous effects of STOMAGEN are local and limited in range. Comparison of stomatal density within sectors, in the nearby regions and in the rest of cotyledons for each sector type revealed that both control vector-only sectors and STOMAGEN sectors did not exhibit statistically significant differences between regions, whereas a gradual decay of EPF1 effects with distance was evident (Fig. 3G).

Next, we expanded the nearby region from 100 μm to within 200 μm (Fig. S3). At this range, the stomatal densities in the nearby region of STOMAGEN sectors and in control empty vectors were no longer statistically different (*P*=0.058) whereas the region of EPF1 remained effective (*P*=0.034) (Fig. S3A,B). Because stomatal density in the rest of the cotyledon outside the 200 μm range remained low for EPF1-overexpressing sectors (Fig. S3B), we tested whether the inhibitory effect of EPF1-overexpressing sectors expands to the entire cotyledon. For this purpose, consecutive bin ranges of 50 μm increments from the sector boundary were set, expanding out to 400 μm. At this range, the effects of the EPF1 sector became negligible (*P*=0.440) (Fig. S3C). Our findings are consistent with the role of EPF1 as secreted peptide inhibiting stomatal development and indicate that both EPF1 and Stomagen have limited effective range. However, because of the rapidly changing heterogeneity of the impact of the peptides on stomatal density in a gradient manner, we conclude that precise quantification of their effective range requires a different metric.

### SPACE analysis quantifies 2D spatial patterning of stomata

Our goal was to determine quantitatively the effective range of peptide signals influencing epidermal patterning. However, currently available and widely adopted quantification methods (stomatal density and index) do not take into account 2D spatial information and can only infer that non-cell autonomous effects exist (Figs 1–3). The stomatal density represents the number of stomata in a given region of interest (ROI) and the stomatal index represents the percentage of stomata in a given number of epidermal cells. With these simplistic parameters, it is not possible to normalize the inherent heterogeneity of mosaic sector size and geometry, which are constrained by the individual size and geometry of epidermal cells constituting GFP_ER_ sectors. Likewise, the exact locations and numbers of individual sectors within a field of cotyledon epidermis is unique to individual heat-shock events. Hence, it is imperative to develop a new technique for quantitative description of stomatal spatial patterning.

**Fig. 4.**
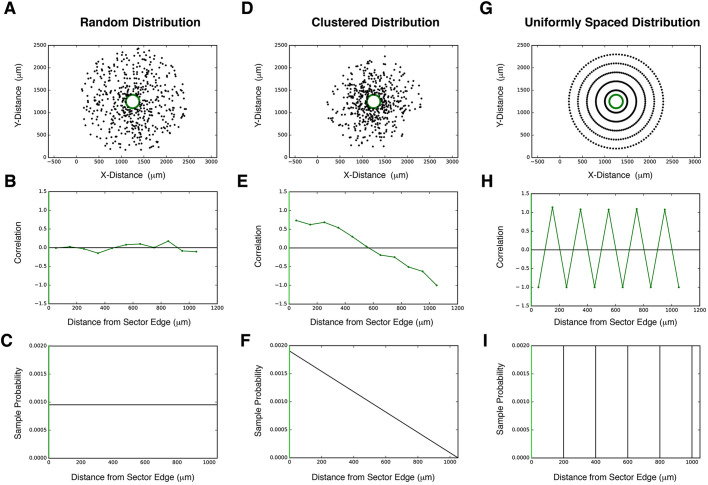
**Simulating 2D spatial patterning with SPACE.** (A-C) Representative example of a uniformly random distribution and its statistical properties relative to a sector. Sample stomata were generated (A; black; *n*=500) relative to a sector (A; sector boundary highlighted in green). Stomata were generated according to the probability distribution in C, and its stomata-sector correlation function in B was calculated directly from the generated points in A (see ‘Materials and Methods’ for calculation). The correlation function is close to zero both near and far from the sector, consistent with a uniformly random probability distribution. The X-axis 0 in B and C corresponds to a sector boundary (green). (D-F) Representative example of a clustered distribution and its statistical properties relative to a sector. Sample stomata were generated (D; black; *n*=500) relative to a sector (D; sector boundary highlighted in green). Stomata were generated according to the probability distribution in F, and its stomata-sector correlation function in E was calculated directly from the generated points in D (see ‘Materials and Methods’ for calculation). The correlation function is highly positive at close distances, consistent with strong clustering of stomata near the sector. The X-axis 0 in D and E corresponds to a sector boundary (green). (G-I) Representative example of a uniformly spaced distribution and its statistical properties relative to a sector. Sample stomata were generated (A; black; *N*=500) relative to a sector (A; sector boundary highlighted in green). Stomata were generated in rings around the sector, each a radius of 200 µm larger than the previous, corresponding to the probability distribution in H. The stomata sector correlation function in I was calculated directly from the generated points in G (see ‘Materials and Methods’ for calculation). As distance increases outward from the sector, the correlation function oscillates between positive and negative, corresponding to regions of stomata (positive) and empty space (negative). The X-axis 0 in H and I corresponds to a sector boundary (green).

To this end, we adapted a statistical technique used by astrophysicists to measure spatial correlation between galaxies at different separations (Landy and Szalay, 1993; Peebles, 1974) (Fig. 4). Stomata are treated as spatial coordinates generated from an unknown probability distribution that determines their spatial patterning (Fig. 4A,D,G). Unlike the probability itself (Fig. 4C,F,I), which cannot be determined from the sample alone, the spatial correlation function can be calculated directly from the stomata coordinates as an accurate and effective approximation of the true probability distribution. The spatial correlation statistic describes this probabilistic distribution of stomata as a function of distance from a sector edge (Fig. 4). If, at a certain distance away from the edge of a GFP_ER_ sector, stomata are more likely to be found than randomly distributed, stomatal production is positively correlated with sector location (Fig. 4E,F). If stomata are less likely to be found than a randomly generated point, stomatal production is negatively correlated with sector correlation (Fig. 4G,H). If stomata production at a distance is as equally likely as random point generation, this implies zero correlation between stomata production and the sector at that range (Fig. 4B,C).

**Fig. 5.**
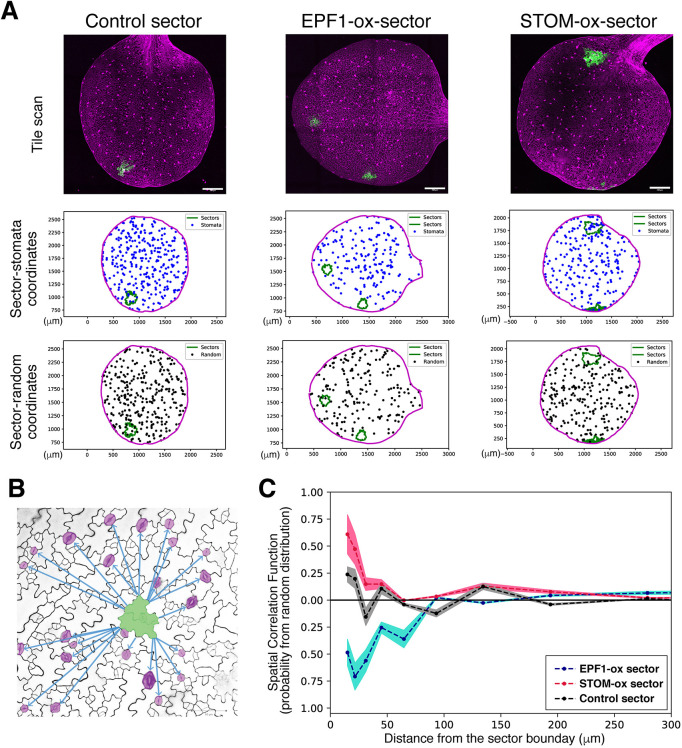
**SPACE analysis determines the effective range of signaling peptides.** (A) Representative data for SPACE pipeline. Top: Representative fully tiled Z-stack confocal microscopy of entire cotyledons with sectors expressing vector-only control (left), EPF1 (middle), and STOMAGEN (right). Middle: Plot of the tiled confocal images. XY-coordinates of cotyledon outlines (magenta), sector outlines (green) and all stomata on the entire cotyledon (blue) are registered. Numbers of stomata in each image: *n*=311 (control), 187 (EPF1), 237 (STOMAGEN). Bottom: One representative plot of the 100 plots of randomly distributed virtual stomata (black dots) with the identical *n* to the actually observed stomata in the images above. (B) Scheme of SPACE analysis. Here, quantitative measurements were performed for the nearest distance between the edge of a sector (green) and every single stoma (magenta) as well as the nearest distance between the edge of a sector and every single random dot (randomly placed virtual stoma) generated computationally (see A, middle). See ‘Materials and Methods’ for calculations. (C) SPACE analysis plot. The autocorrelation of sector to stomata in the function of distance from the sector boundary. Control sector autocorrelation (gray) exhibits subtle peaks at proximity ∼50 µm and at ∼150 µm, which may correspond to two stomata separated by the one-cell spacing rule and secondary spacing. The STOMAGEN-ox sector (red) exhibits a strongly positive correlation at the sector boundary, which decays within the first ∼60 µm. By contrast, EPF1-ox sector (blue) exhibits a negative correlation that gradually decays at around 100-150 µm. Colored area represents 95% confidence range. Scale bars: 250 µm.

To calculate spatial correlation, we plotted the 2D positions (XY coordinates) of every single stoma on an entire cotyledon, plus sector outlines and cotyledon outlines from each full tile-scanned confocal Z-stack images of an entire cotyledon (Fig. 5A, top and middle). To compare, we computationally generated 1000 sets of random point distributions of ‘dummy’ stomata, which were exactly the same total numbers as that of the ‘real’ stomata within the identical cotyledon outline (Fig. 5A bottom; see ‘Materials and Methods’). The nearest Euclidan distance was calculated between each stoma and the edge of a sector outline, excluding stomata inside the sector, and for every random set, the nearest Euclidean distance was calculated between each random point and the edge of a sector outline. (Fig. 5B; see ‘Materials and Methods’). After repeating this process for 1000 equally sized sets of random points per cotyledon, the aggregate distribution of distances between random points and sectors approached its expected probability and allowed us to calculate stomatal spatial correlation as a function of distance (see ‘Materials and Methods’ for further details and calculations). The spatial autocorrelation statistics register the nearest distance of every single stoma from the sector edge and compare it with the random point sets of equal numbers to actual stomata. These random points are placed within the exact same cotyledon outlines and analysis is reiterated 1000 times. Because of the strict cotyledon-wide analysis of all stomata, the SPACE pipeline enables quantification of changes in stomatal distribution at different distances relative to GFP_ER_ sectors, independently of leaf shape, leaf size, sector placement or sector size in a way that consecutive bin range analysis of stomatal density (Fig. S3C) cannot. Furthermore, the magnitude of spatial correlation quantifies the degree of change in stomatal distribution, allowing us to measure how the influence of peptide overexpression changes with distance.

### EPF1 and Stomagen differ in effective range

The SPACE analysis generated a probabilistic distribution of stomata as a function of distance from the sector boundary (Fig. 5). Control empty-vector-only sectors did not show substantial positive or negative correlations but exhibited fluctuations within short ranges. A fluctuation of negative correlation in between two positive peaks may reflect a one-cell spacing rule enforced between two stomata located at the 10 and 50 µm peaks, whereas the subtle drop and re-gain of correlation from 50 to 150 µm may suggest a secondary spacing caused by the presence of a stomata at around 50 µm (Fig. 5C). At close distances, stomatal production was negatively correlated with EPF1 sectors, positively correlated with Stomagen sectors and uncorrelated with control empty-vector sectors (Fig. 5C). At farther distances, spatial correlation between stomata production and peptide overexpression approached zero for both EPF1 and Stomagen, which corresponded to the effective range of the peptides. These results suggest that the spatial correlation method can quantify our visual observations from the tile scans. Sectors of EPF1 overexpression exhibit an effective range of >100 µm and sectors of Stomagen overexpression an effective range of ∼60 µm. Initially, we introduced sector size filters (15,000-40,000 µm^2^) to the pipeline to avoid deviation due to sector size, geometry or interference if multiple sectors exist on the same cotyledon. Removing the sector size filters (no maximum limit) did not essentially change the spatial autocorrelation (Fig. S4), thus revealing the robustness of SPACE analysis to sector size, geometry and numbers. Combined, the results suggest that production of EPF1 is capable of affecting stomatal development at farther ranges than Stomagen. Furthermore, the absence of discernable effects beyond 300 µm implies that local manipulation of stomatal patterning by sector-limited overexpression of EPF-family peptides does not influence global stomatal patterning throughout the cotyledon.

## DISCUSSION

Members of the EPF and EPFL family of peptides regulate stomatal development at distinct stages to enforce proper spacing across the epidermis. Our study establishes the extent of the non-cell-autonomous capability of EPF1 and Stomagen to influence stomatal patterning. To compare the effective range of these two peptides with antagonistic activities on stomatal development, we used the ectopic Cre-lox and Gal4/UAS system to address how far EPF1 and Stomagen can influence stomatal patterning when expressed at equivalent levels in a similarly confined area (i.e. mosaic sectors). It should be noted that the mosaic analysis does not reflect endogenous expression levels and patterns, which contribute to normal ‘wild-type’ stomatal patterns. Nevertheless, our detailed analysis of stomatal index and density clearly demonstrate the non-cell-autonomous nature of these peptides, with a clear decay of effect with distance and cell number. To address the limitations of standard phenotypic analyses, such as stomatal index and density, we created a computational pipeline, SPACE, to apply a correlation-based spatial point analysis and quantify precisely the effective ranges of action of the peptides.

### Correlation-based approaches in stomatal patterning

There is a growing need to quantify stomatal patterns at higher spatial resolution because stomatal mutants may have similar densities but different patterning, as underlied by distinct molecular mechanisms. A common measurement used to address this, in addition to the stomatal index, is a count of stomata clusters that violate the one-cell spacing rule, thus extending stomatal index to clustering index or histograms to display their distributions. Our analysis of stomatal density across mosaic-cotyledons indicates that the reduction in stomatal density due to the presence of EPF1 sectors decayed with distance, and that the impact of EPF1 on the stomatal phenotype acted as a gradient. The rapidly changing heterogeneity of the impact of the peptide on patterning makes not only stomatal density a limited approach, but also the most common extensions to counting statistics such as the aforementioned cluster index. Thus, although we established the existence of an EPF1 effective range and a gradient in stomatal phenotype, quantifying their values with precision necessitated a different metric, the SPACE.

Statistical methods of spatial point analysis from other fields have begun to be embraced, and it is vital to find the specific metric suitable for extracting the information each individual study needs. One such technique is the use of Betti numbers from persistent homology (Haus et al., 2018). Applied to stomatal patterning, the 0th Betti number counts the number of stomata clusters (called ‘components’) across a leaf that remain separate when stomata are connected by a radius of given distance. Increasing the radius gradually decreases the total number of connected components until they eventually merge into a single set, allowing one to see how the topology of stomatal distribution changes across varying spatial resolutions. To elucidate how local overproduction of stomatal peptides impacts patterning, it is necessary to utilize an algorithm that can determine the strength of disruption as a function of distance from a particular location (the sector), rather than overall connectedness of the pattern. It would also be difficult to interpret Betti numbers for *EPF1*-overexpressing mosaics in particular, as there are little to no stomata in or near the sectors to connect to, no matter the distance. Therefore, we require our choice of metric to measure pairwise interactions and to have a bivariate form applicable to two separate point distributions: the sector outline and the stomata.

The greater correlation length of EPF1 compared with that of Stomagen may come as a surprise considering that, in wild type, STOMAGEN is first expressed in the subepidermal tissue, which eventually differentiates into the mesophyll before secreting to influence stomatal patterning in the epidermis. By contrast, EPF1 expression is in the epidermis itself. From the perspective of biochemical properties, although the structures of Stomagen and EPF peptides have been resolved (Lin et al., 2017; Ohki et al., 2011), nothing is known about the actual diffusion capacity of EPF/EPFL-family peptides nor about specific modifications that determine their effective range. Interestingly, the loop domains of EPF1 and Stomagen are structurally distinct, with EPF1 having an additional disulfide bond within the loop domain, which might provide stability (Ohki et al., 2011). A domain-swap analysis has shown that the loop domain governs the biological activities of Stomagen and EPF2 as an activator and inhibitor of stomatal development (Ohki et al., 2011). However, there is no experimental evidence demonstrating that the loop domain contributes to the effective action range of each peptide.

In the context of an activator-inhibitor system, such as in Turing patterns, it is necessary that the inhibitor has a longer range than the activator, as observed in this study, and that the activator induces both the inhibitor and itself (Kondo and Miura, 2010; Meinhardt, 2012). Although Stomagen does not directly induce EPF1, it leads to generation of cell types (stomatal precursors) that express EPF1. Thus, the difference in their effective ranges might imply some universal theme of activator-inhibitor relationships in biological patterning. The effective ranges of EPF1 and Stomagen determined by the SPACE pipeline roughly correspond to three stomata and two pavement cells, respectively. Although future studies are needed, these results emphasize the idea that EPF and EPFL peptides act as short-range signaling peptides. Before interpretation, it must be noted that the correlation or anticorrelation distance of a peptide, also describable as an effective range of action, is not equivalent to an actual distance of diffusion. Mechanisms that contribute to an effective range of action also include the threshold of concentration each peptide must have within a cell to change a cell fate decision, the geometry of cell expansion (e.g. pavement cell geometry) and potential regulatory feedback loops. For instance, clusters of stomata produced by a Stomagen sector might produce and secrete EPF1 to cells further away, buffering against Stomagen overexpression. Further studies are required to elucidate the degree to which these individual mechanisms contribute to the measured correlation lengths and amplitudes.

In any event, this quantification approach enables each of these mechanisms to be viewed as a variable that fine-tunes the correlation function. Different features of the matter correlation function enable physicists to study the mechanism that dominates that region of the function, such as gravity or baryonic acoustic oscillations (Cole et al., 2005; Eisenstein et al., 2005). Analogously, different features of a stomatal correlation function may correspond to specific genes or mechanisms in stomatal patterning. Our SPACE pipeline is not limited to the context of stomatal development; it could be utilized for quantitative analyses of phenotypic characteristics and mathematical constraint, and broadly to the study of spatial patterns of individual cell fate, such as floral spot patterning (Ding et al., 2020).

### Local and global patterning in stomatal development

It has been reported that Stomagen as well as EPFL4 and EPFL6/CHALLAH are expressed in non-epidermal tissues, but they could modulate stomatal patterning (Abrash et al., 2011; Kondo et al., 2010; Sugano et al., 2010; Uchida et al., 2012). Consistent with these findings, our study identified EPF1-expressing sectors exclusive to the mesophyll that still inhibited stomatal development in the nearby epidermis (Fig. 2). Combined, these results highlight the necessity of viewing stomata development as a multidimensional system that acts and coordinates across multiple tissues. EPF peptides may play a key role in the inter-tissue communication between stomata that mediate gas exchange and the photosynthetic mesophyll. A previous study emphasized the crucial functions of stomatal patterning signal transduction pathways mediated by EPF1/2, Stomagen, TMM and ERECTA-family receptor kinases on mesophyll development: loss-of-function or overexpression of these signaling components influence mesophyll cell density and photosynthetic potential (Dow et al., 2017). A more recent study has shown the importance of mature functional stomata and actual gas exchange for mesophyll air-space morphogenesis (Lundgren et al., 2019). Thus, inter-tissue-layer communication involves peptide signaling at an early developmental stage and mechanical/physiological feedback during maturation. The expression of EPF peptides in internal tissues also raises the question of stomatal signaling between the abaxial and adaxial sides of the leaf. In future studies, the correlation in stomata positioning between the abaxial and adaxial stomata on the same cotyledon could be measured.

Previous studies have shown the presence of long-range hormone signaling that acts on stomatal development (Casson and Gray, 2008; Qi and Torii, 2018). In this study, we developed a pipeline that enables the quantitative measurement of spatial correlation and density at different scales of distances, separating local and global features of stomatal patterning and production. Our SPACE analysis could be used to address whether local manipulation of stomatal development could in turn influence the global stomatal patterns. For instance, locally upregulated EPF1 or Stomagen signaling could impinge on longer range hormone signaling, such as via auxin, to induce a compensatory increase or decrease in stomatal development globally. In fact, auxin and EPFL2 peptide signaling pathways constitute negative feedback during leaf morphogenesis (Tameshige et al., 2016). By contrast, we observed that in the epidermal tissue defined as far away from Stomagen-expressing or EPF1-expressing sectors, stomatal patterning returned to normal both in density and in correlation (Fig. 3D-G, Fig. 5C; Fig. S1, Fig. S3). The lack of evident compensation could have several possible explanations. For instance, local manipulations of small EPF1- or Stomagen-expressing sectors are not sufficient to trigger an above-threshold compensatory response. It has been reported that the overall mechanical properties of leaf epidermis could impact the polarity of stomatal lineage cells (Bringmann and Bergmann, 2017). Secondary changes in stomatal signaling as a result of sector overexpression might ‘buffer’ the global influence. Our system might be more applicable for studying the global ripple of local perturbations in mature leaves, as physiological feedback increases in importance. With the pipeline to detect and quantify local versus global patterns in hand, future studies of mechanical and physiological feedbacks will provide a comprehensive picture of stomatal development in the context of a whole functional leaf.

## MATERIALS AND METHODS

### Plant materials and growth conditions

*Arabidopsis thaliana* Columbia (Col-0) accession was used as wild type. The two-component Cre-lox Gal4/UAS system was reported previously (Heidstra et al., 2004). Transgenic lines were generated by genetic crosses or Agrobacterium-mediated transformation into the Col-0 background; genotypes were confirmed through PCR. Seeds were sown on 0.5× Murashige and Skoog (MS) medium containing 1× Gamborg Vitamin (Sigma), 0.75% Bacto Agar, and 1% sucrose. After stratification at 4°C for 2 days, seeds were grown in long-day condition at 21°C. To generate mosaic sectors, at 24 h after gemination seedlings were subjected to heat-shock treatment in a 37°C incubator as described below.

### Molecular cloning and generation of transgenic plants

The two component Cre-lox system (Heidstra et al., 2004) was modified to express full-length EPF1, EPF2 and *STOMAGEN*. *EPF1* cDNA from pTK106 (Lee et al., 2012) was digested with BamHI and EcoRI and ligated into pBnUASPTn to generate pTK109. *STOMAGEN* cDNA was amplified by PCR using a plasmid pTK129 as a template and cloned into pCR2.1 TOPO vector (ThermoFisher/Invitrogen) to generate pJS104 and the sequence confirmed. Subsequently, the insert was ligated into pBnUASPTn, a transient vector to clone cDNA of interest under Gal4 UAS, to generate pJS105. These constructs were digested by NotI and ligated into pGII277-HSCREN2 vector, which contains heat-shock promoter-driven CRE recombinase gene, to generate pTK111, pTK112 and pJS106. These three plasmids and pCB1, which carries 35S::lox-CRT1-lox: Gal4-UAS-GFP_ER_ (Heidstra et al., 2004), were individually transformed into *Agrobacterium* GV3101 (pMP90) in the presence of pSOUP (Hellens et al., 2000). Subsequently, *Arabidopsis* plants were transformed by a floral dipping method. More than 48 T1 plants were characterized. Three lines each of pTK111, pTK112 and pJS106 with a monogenic inheritance of selection markers were subjected to genetic crosses with the pCB1 lines, and two or three lines were chosen for further analysis based on the heat-shock inducibility of the *Cre* transgene as well as formation of chimeric sectors (see below). As a control, pGII277-HSCREN2 vector was transformed into pCB1 transgenic line. Table S2 lists plasmids used in this study and Table S3 lists primer sequences used for molecular cloning and genotyping of the transgenes.

### Heat-shock induction and sector identification

To generate mosaics, seeds after 4°C stratification were grown in long-day conditions at 21°C. At 24 h after germination, seedlings were heat-shocked in a 37°C incubator. We first tested different durations of heat-shock treatment and optimized the resulting GFP+ sector size and number. Heat-shock treatment lasted 15 min to generate mosaics used in imaging experiments and 1 h to generate mosaics used in qRT-PCR experiments. Prior to imaging experiments for 7-day-old seedlings or qRT-PCR experiments for 5-day-old seedlings, as detailed below, seedlings with mosaic overexpression were identified using a dissecting microscope equipped with GFP fluorescence detection (Leica M165FC).

We initially sought to include EPF2, an EPF/EPFL-family peptide restricting the initiation of stomatal development, to our pipeline. However, EPF2 sectors failed to show discernable effects. It is well established that EPF2 acts on the initial stomatal lineage entry (Hara et al., 2009; Hunt and Gray, 2009; Lee et al., 2012). In our detailed time-lapse analysis of stomatal cell-state transition during germinating cotyledon development (Han et al., 2018; Peterson and Torii, 2012), the initial commitment to stomata, as monitored by the onset of MUTE protein accumulation, occurs ∼30 h after germination. After extensive optimization, we determined that heat-shock treatment given 24 h after germination is the best condition for reliably generating reproducible size and number of sectors. This means that while the Cre-lox recombination is producing EPF2-overexpressing sectors, the initial EPF2-regulated stomatal lineage transition has already passed. A review of available literature, our time-lapse analysis of detailed stomatal-lineage marker expression during germination, and our heat-shock Cre-lox experiments emphasize the strict developmental time window of EPF2 action, which is too early to address because of our technical limitations. As a result, we did not see statistically significant inhibition of stomatal development by EPF2-overexpressing sectors. Owing to the early events of EPF2-mediated repression of stomatal initiation during seedling germination (1-2 days) (Hara et al., 2009), the timeframe of heat-shock-induced recombination and mosaic overexpression was too late to induce clear effects. For this reason, EPF2 sectors were not subject to further analysis.

### Reverse-transcription PCR

Five-day-old seedlings treated with heat-shock as described above were subjected to RNA preparation using RNAeasy kit (Qiagen). Subsequently, cDNA was synthesized using the iScript cDNA synthesis kit (Bio-Rad) according to instructions of the manufacturer. First-strand cDNA was diluted to one seventh in double distilled water and used as template for qRT-PCR. Quantitative RT-PCR was performed as described previously (Han et al., 2018) with a CFX96 real-time PCR detection system (Bio-Rad) using iTaq SYBR Green Supermix with ROX (Bio-Rad). Relative expression was calculated by normalizing *ACT2* gene expression over the specific gene expression. For each experiment, three technical replicates were performed. RT-PCR was performed as described previously (Lee et al., 2012). See Table S2 for a list of primer sequences.

### Microscopy and tile scan analysis and quantification

Confocal laser scanning microscopy images of 7-day-old seedlings were taken with the Zeiss-LSM700 (Zeiss) or the Leica SP5-WLL (Leica). Cell peripheries were visualized with propidium iodide (PI; Molecular Probes, Carlsbad, CA). GFP and PI signals were detected with excitation at 488 nm and 555 nm, respectively, and emission at 500-524 nm and 569-652 nm, respectively. The tile scan images were analyzed using Imaris 9.2 (Bitplane) as follows. First, GFP-expressing 3D sectors were segmented using the Surface function by thresholding the absolute intensity of the green channel, with background autofluorescence subtracted afterward. Next, sector outlines, the full cotyledon outline and the stomatal positions were recorded with 3D voxel spatial coordinates using the Spots function. These spatial coordinates were then exported to Microsoft Excel v.16.32 as .xlsx workbooks (Microsoft), then converted to .xlsx format for quantitative 2D spatial analysis as detailed below. To identify GFP sectors generated exclusively in the mesophyll, 3D images of sectors were analyzed in the XZ and YZ planes using Leica Application Suite AF's Orthogonal View, as well as in three spatial dimensions using Imaris 3D volume rendering. Mesophyll-exclusive sectors were segmented and their positional outlines marked using the same procedure as described above. For figure preparation, the brightness and contrast of images were uniformly adjusted (increasing by 20) using Photoshop CC (Adobe). For cell size measurement, ImageJ was used to quantify the longest and shortest sides of stomata and non-stomatal pavement cells and the one-cell spacing between two given stomata.

### Geometric sectors for heat-shocked wild-type cotyledons

In addition to empty vector GFP sectors (control sectors), sector outlines were overlaid onto heat-shocked wild-type cotyledons as another control (geometric sectors). To generate geometric sectors, the coordinates recorded from the outlines of real mosaic GFP-expressing sectors were overlaid onto heat-shocked wild-type cotyledons. Sector outlines were transposed onto wild-type images without bias by randomly generating a shift to the center of the sector outline before plotting it on the image. If part of the newly transposed sector fell outside the boundary of the wild-type cotyledon outline, it was excluded from stomata quantification analysis. To calculate the stomatal index inside and nearby geometric sectors, any cell with at least half of its area contained inside the sector outline was considered part of the geometric sector.

### Quantitative two-dimensional spatial analysis

Spreadsheets of stomatal coordinates, sector outline coordinates and cotyledon outline coordinates, recorded in three dimensions as described above, were processed and analyzed using SPACE (stomata patterning auto correlation on epidermis), a pipeline of Python scripts written by us (available at https://github.com/ToriiLab/CreLox). Stomata, sectors and cotyledons were plotted, visualized and analyzed two dimensionally using their XY coordinates. To determine the stomatal density of a cotyledon within GFP sectors, our script calculates the number of stomata inside a sector outline and the area enclosed by the outline. For cotyledons with multiple sectors of GFP expression, the stomata counts and sector areas were aggregated before calculating the overall stomatal density of the cotyledon within sectors as a single sample point.

To calculate stomatal density within a 100 µm range of sectors, a new outline was generated by applying a 100 µm radially outward shift to each sector outline coordinate. Stomata and epidermal areas enclosed by the new outlines were then calculated to find the stomatal density in this region, excluding the region enclosed by the original sector outlines and any region extending beyond the cotyledon outline. For cotyledons with multiple sectors, calculations were made for the union of the new outlines, to avoid counting overlapping regions multiple times. This same process was then repeated for a 200 µm range as well as a consecutive bin range of 50 µm increments up to 400 µm.

To calculate the spatial correlation function between stomata positions and sector outlines, the nearest Euclidean distance was calculated between each stoma and the edge of a sector outline, excluding stomata inside a sector. The same process was repeated 1000 times, each with independently generated random point distributions within the cotyledon outline, each equal in size to the total number of stomata across the leaf. Random point distributions were generated by first producing five times in excess the number of stomatal points within a rectangle, then running a function to keep only the random points lying within the cotyledon outline, and finally only keeping the first *N* points in a list, where *N* was the same as the number of actual stomata. Distances between sectors and stomata, and distances between sectors and random points for each independent distribution, were counted in histograms of logarithmically spaced bin widths. The spatial correlation function ζ between stomata positioning and sector location was calculated using the bivariate extension of the two-point correlation function in astronomy (Landy and Szalay, 1993; Peebles, 1974), also known as the differential form of the Ripley's *K* function (Ripley, 1976):(1)
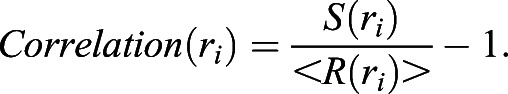


*S*(*r*_i_) is the number of stomata counted between a distance of *r*_I_ and *r*_i+1_ away from a sector. <*R*(*r_i_*)> is the expected value of the number of random points counted between a distance of *r*_I_ and *r*_i+1_ away from a sector, estimated by averaging the number of points counted in that range of distance for 1000 random distributions. Because sector size might influence the range and magnitude of correlation, our code filters sectors so that only those within a range of area are analyzed. To avoid cross-correlations on cotyledons with multiple sectors, this analysis is also filtered for sectors located within 200 µm of each other on the same cotyledon, also filtering by sector size (15,000-40,000 µm^2^) (see Fig. 5). To address whether filtering changes the effective ranges, SPACE analysis was also performed without sector size filtration (see Fig. S4). Confidence intervals were obtained via resampling techniques.

### Stomata-stomata autocorrelation function

To quantify the autocorrelation of stomatal patterning with itself, the Euclidean distance between each unique pair of stomata, for all stomata, across a cotyledon, was calculated. As described above, the same process was repeated for 1000 independently generated random point distributions within the cotyledon outline, each equal in size to the total number of stomata across the cotyledon. Distances between pairs of stomata, distances between pairs of random points within a given distribution and distances between stomata and random points of a given distribution, were counted in histograms of logarithmically spaced bin widths. The stomata autocorrelation function was calculated using the following estimator function used in astrophysics to minimize bias and variance of the two-point galaxy autocorrelation function (Landy and Szalay, 1993; Peebles, 1974):(2)
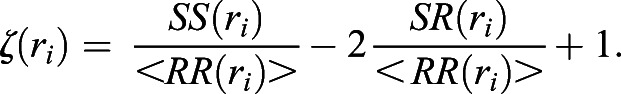


*SS*(*r*_i_) is the number of stomatal pairs counted that are separated by a distance between *r*_I_ and *r*_i+1_*. RR*(*r*_i_) is the number of random point pairs within one distribution counted that are separated by a distance between *r*_I_ and *r*_i+1_. *SR*(*r*_i_) is the number of distances separating a stoma and a random point between *r*_I_ and *r*_i+1_.All three are normalized by the total number of pairs for that variable. <  > indicates expected value and is estimated by averaging across 1000 randomly generated distributions.

### Statistics

Graphs were generated using R ggplot2 package or Python matplotlib. All scripts are available (GitHub account). A chi-squared test for statistical independence on 2×2 contingency tables was used for comparing the stomatal index between sectors of different peptide overexpression, in order to determine whether there was a statistically significant difference between the ratio of stomata to epidermal cells in one population versus another. For quantitative analysis of stomatal index and stomatal density in individual sectors of different genotypes, box plots were generated with individual data points overlaid as jitters. For comparison of individual sector data, Welch's two sample *t*-test was performed for pairwise comparison between control and other genotypes (geometric, EPF1-overexpressing and Stomagen-overexpressing). The Mann–Whitney *U-*test was used for comparing stomatal densities, as we did not assume that stomatal density was normally distributed among the population.

## Supplementary Material

Supplementary information

Reviewer comments
