# Supplementary material for: Effective range of non-cell autonomous activator and inhibitor peptides specifying plant stomatal patterning
Source: Development. 2020 Sep 11;147(17):dev192237. doi: 10.1242/dev.192237 (PMC7502594; doi:10.1242/dev.192237)
Supplement: Supplementary information [file develop-147-192237-s1.pdf]

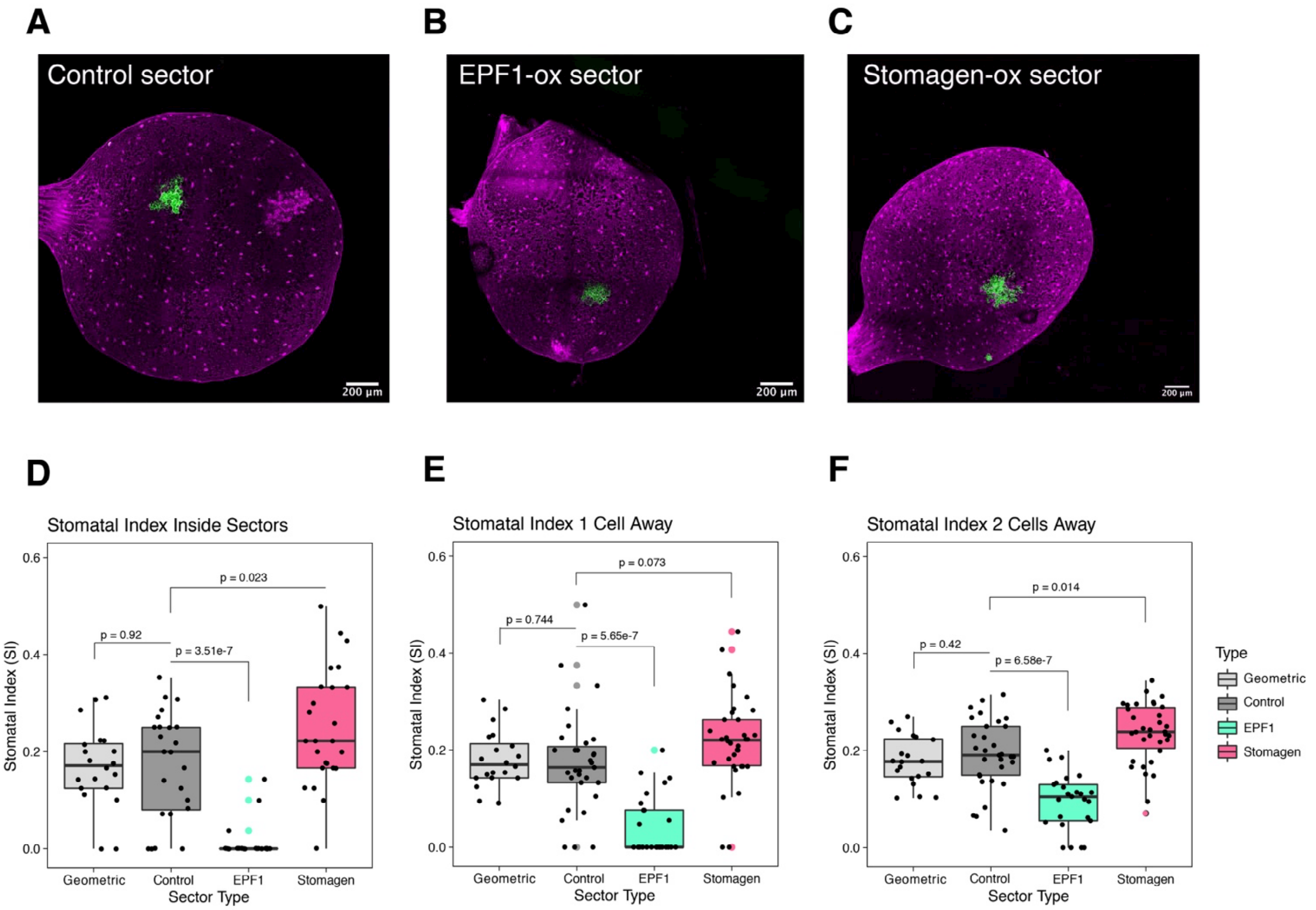

Fig. S1: Full tile scan images of mosaic seedlings and individual sector-based stomatal index analysis (Related to Figure 1)

(A) Cotyledon with a control sector, scale bar = 200 μm

(B) Cotyledon with an EPF1-overexpressing (EPF1-ox) sector, scale bar = 200 μm

(C) Cotyledon with a Stomagen-overexpressing (Stomagen-ox) sector, scale bar = 200 μm.

(D-F) Stomatal Index within sectors (D), cells immediately adjacent to sectors (E), and cells adjacent to immediate neighboring cells (F) For wild type, virtual geometric sectors (geometric) of the same size and geometry as real sectors were computationally placed. Individual sectors were analyzed separately without data aggregation. Number of sectors subjected to analysis; n=20 (geometric), n=34 (control), n=25 (EPF1), n=31 (Stomagen). Box plots were generated for geometric (light grey), control (dark grey), EPF1-ox (lime green), and Stomagen-ox (fuchsia), and individual data points (black dots) are jittered. Grey dots, lime green dots and fuchsia dots represent outliers (Q3 + 1.5*IQR) for control, EPF1-ox, and Stomagen-ox sectors, respectively. For within sector analysis, sectors composed of < 3 cells were uniformly excluded from analysis, as extreme fluctuation of SI (i.e. either 0 or 1) makes the variance among individual sectors too high for statistical analysis. Welch's two-sample t-test was performed for pairwise comparison with control sector data.

Development • Supplementary information

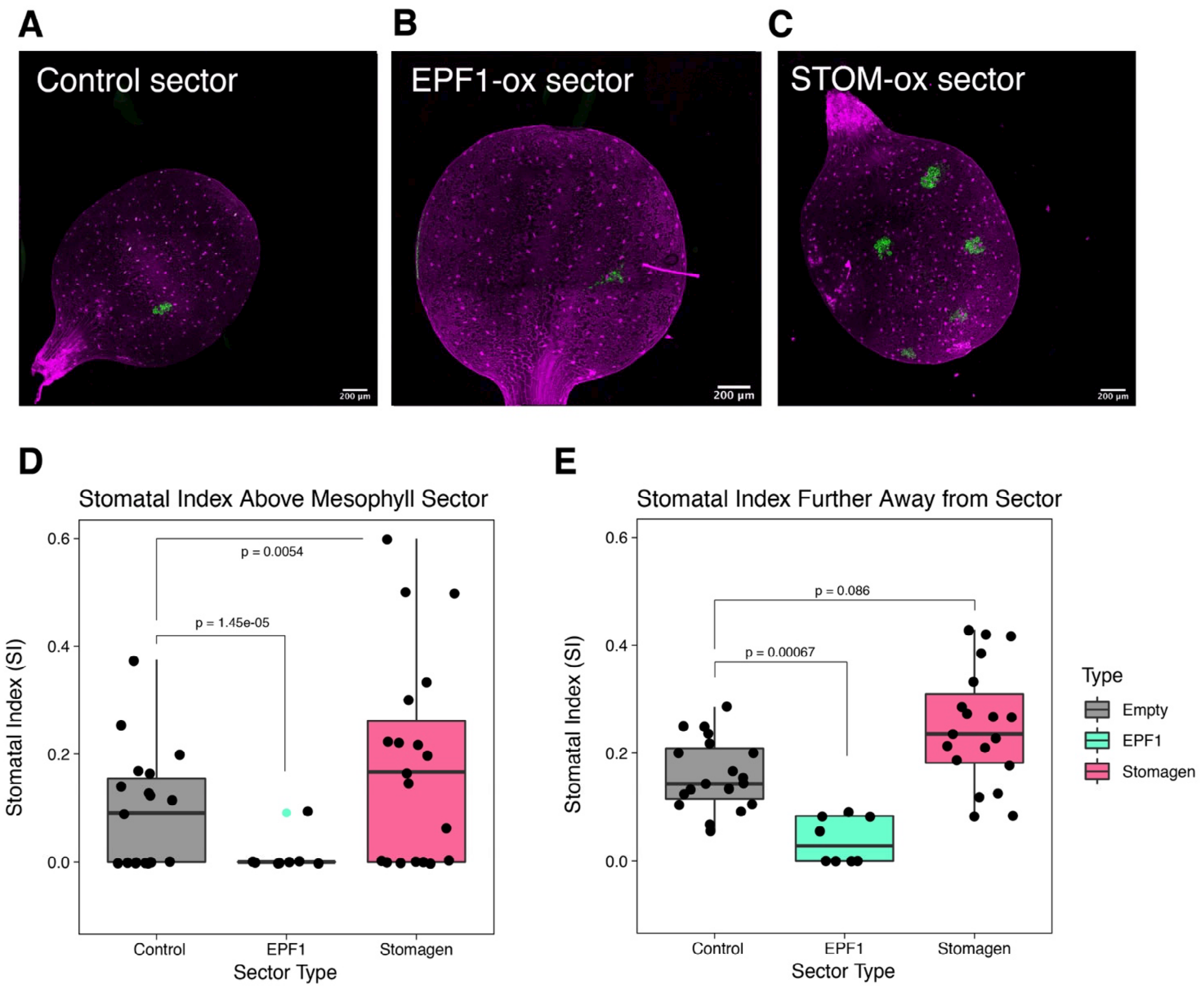

Fig. S2. Full tile scan images of mosaic seedlings and individual sector-based stomatal index analysis (Related to Figure 2)

(A) Cotyledon with a control sector

(B) Cotyledon with an EPF1-overexpressing (EPF1-ox) sector

(C) Cotyledon with a Stomagen-overexpressing (STOM-ox) sector

(D and E) Stomatal Index immediately above mesophyll sectors (D) and further away from sectors (E). Individual sectors were analyzed separately without data aggregation. Number of sectors subjected to analysis; n=19 (control), n=8 (EPF1), n=19 (Stomagen). Box plots were generated for control (dark grey), EPF1-ox (lime green), and Stomagen-ox (fuchsia), and individual data points (black dots) are jittered. Lime green dots represents an outlier (Q3 + 1.5*IQR) for EPF1-oxsector. Welch's two-sample T-test was performed for pairwise comparison with control sector data.

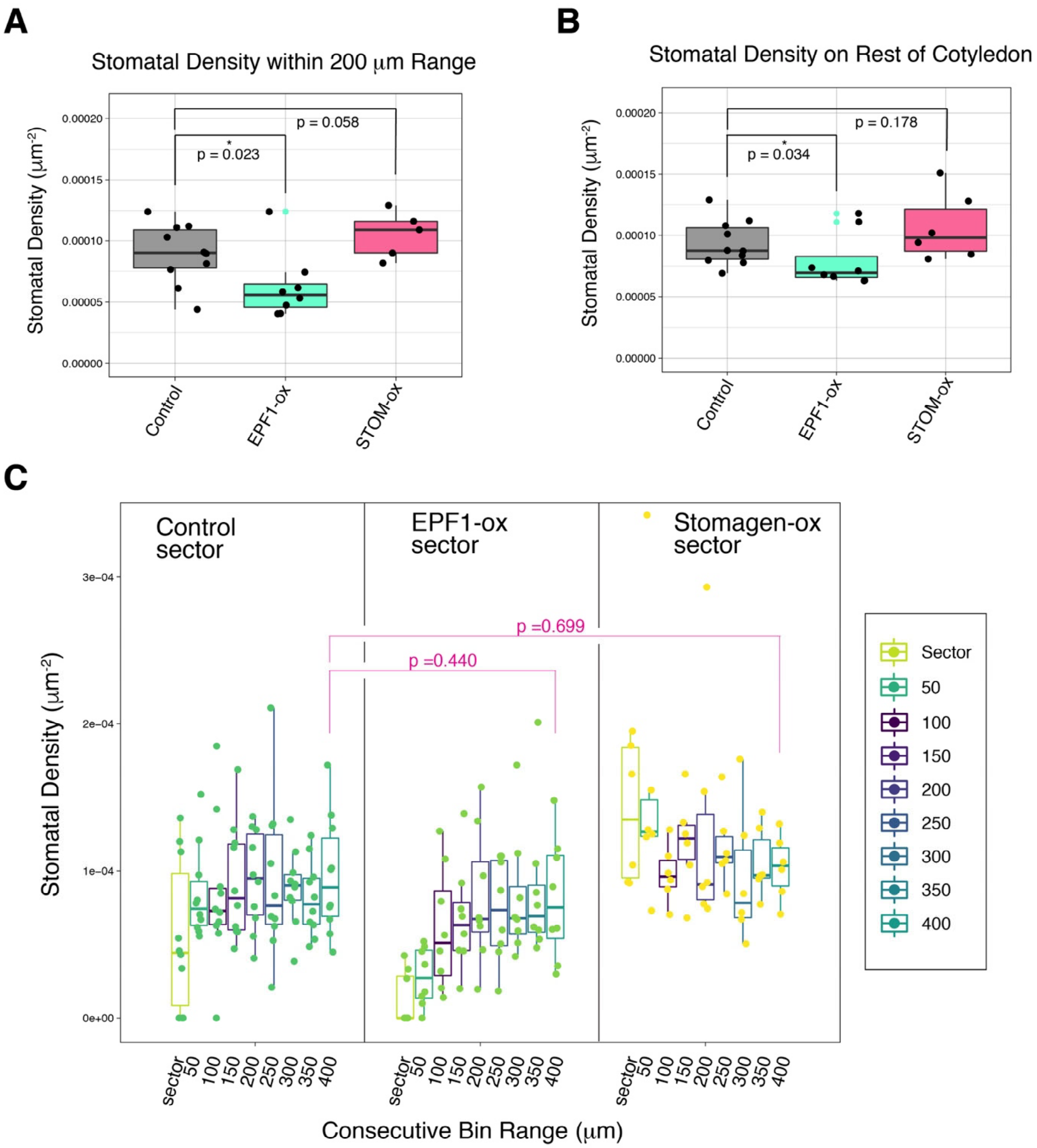

Fig. S3. Quantitative analysis of effective range at 200 μm from EPF1 and Stomagen-overexpressing sectors

(A) Stomatal density within 200 μm Range of sectors for individual cotyledons: cotyledons analyzed are same as in Figure 3. Jitter dots on the boxplot correspond to stomatal density from individual sectors. Total number of stomata counted within 200 μm. Range, n=298 (control); n=176 (EPF1-ox); n=351 (STOM-ox). A Mann-Whitney U test was performed to test significant deviation between distributions of stomatal density.

(B) Stomatal density outside of the 200 μm Range in (A) for individual cotyledons: cotyledons analyzed are same as in (A). Jitter dots on the boxplot correspond to stomatal density from individual

Development • Supplementary information

sectors. Total number of stomata counted on the remaining area of cotyledons, n=2047 (control); n=1641 (EPF1-ox); n=1103 (STOM-ox). A Mann-Whitney U test was performed to test significant deviation between distributions of stomatal density.

(C) Consecutive bin range approach shows the decay of non-cell autonomous effects of EPF1 and Stomagen. Stomatal density are determined within sectors as well as consecutive bin range of 50 μm increment for control sectors (left), EPF1-ox sectors (middle), and Stomagen-ox sectors (right). Jitter dots on the boxplot correspond to stomatal density from individual sectors. Like Stomagen-ox, the effects of EPF1-ox are no longer statistically significant at 400 μm range. Welche's two sample t-test was performed for a pairwise comparison.

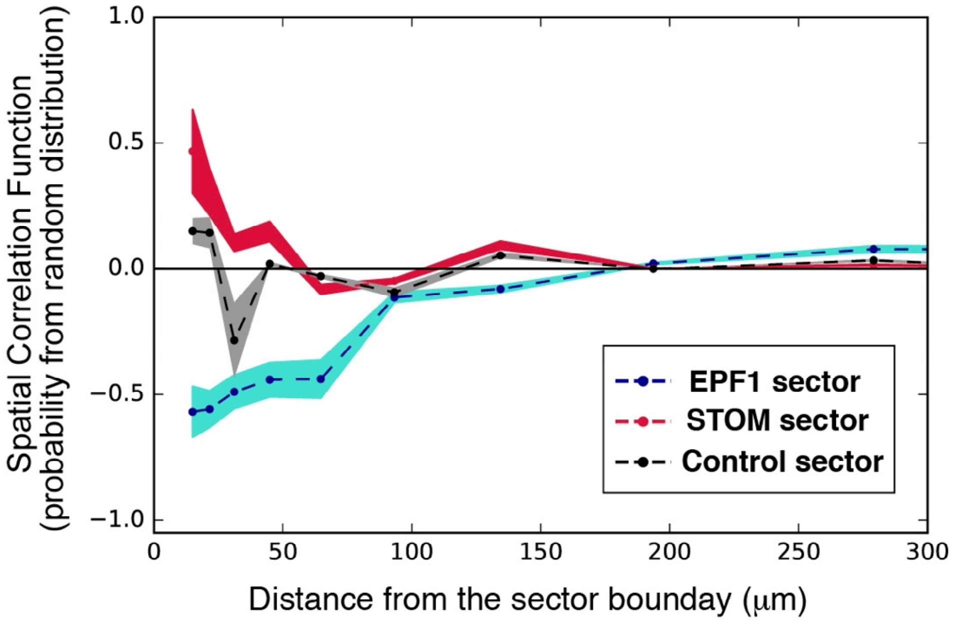

Fig. S4. SPACE analysis without sector-size filtering (Related to Fig. 5)
SPACE analysis plot. The autocorrelation of sector to stomata in the function of distance from the sector boundary. The analysis was performed without filtering sector size (see Methods). Control sector autocorrelation (gray) becomes negatively correlated at the sector junction but exhibits subtle peaks at proximity ~50 μm and at 130~150 μm. The STOMAGEN-expressing sector (red) exhibits a strongly positive correlation at the sector boundary, which decays within the first ~60 μm. By contrast, EPF1-expressing sector (blue) exhibits a negative correlation that sharply decays at around ~100 μm and gradually decays toward ~150-200 μm range. Colored area represents 95% confidence range.

Table S1. Frequency of Genetic mosaics per seedlings

Purpose	Name	Line	Total # Seedlings	# Mosaic Seedlings	Proportion Mosaic
Control	pGII227-HSCREN2 in pCB1-7.1	3	87	42	0.482758621
Control	pGII227-HSCREN2 in pCB1-7.1	4	36	13	0.361111111
Control	pGII227-HSCREN2 in pCB1-7.1	5	42	19	0.452380952
Control	pGII227-HSCREN2 in pCB1-7.5	1	6	4	0.666666667
Control	pGII227-HSCREN2 in pCB1-8.7	1	8	8	1
Control	pGII227-HSCREN2 in pCB1-8.7	2	35	26	0.742857143
Control	pGII227-HSCREN2 in pCB1-8.7	3	6	3	0.5
Control	pGII227-HSCREN2 in pCB1-8.7	6	6	4	0.666666667
EPF1	pTK112-1 x pCB1-8	3	12	7	0.583333333
EPF1	pTK112-1 x pCB1-8	4	4	1	0.25
EPF1	pTK112-1 x pCB1-8	5	8	3	0.375
EPF1	pTK112-1 x pCB1-8	9	6	3	0.5
EPF1	pCB1-7 x pTK112-4	3	6	1	0.166666667
EPF1	pCB1-7 x pTK112-4	4	6	4	0.666666667
EPF1	pCB1-7 x pTK112-4	6	6	2	0.333333333
EPF1	pCB1-7 x pTK112-4	7	7	2	0.285714286
EPF1	pCB1-7 x pTK112-4	9	6	4	0.666666667
EPF1	pTK112-1 x pCB1-8	3.3	48	6	0.125
EPF1	pTK112-1 x pCB1-8	4.1	90	32	0.355555556
EPF1	pTK112-1 x pCB1-8	4.5	20	2	0.1
EPF1	pTK112-1 x pCB1-8	9.1	6	1	0.166666667
EPF1	pCB1-7 x pTK112-4	6.2	12	2	0.166666667
EPF1	pCB1-7 x pTK112-4	6.6	39	8	0.205128205
EPF1	pCB1-7 x pTK112-4	6.8	29	10	0.344827586
EPF1	pCB1-7 x pTK112-4	6.3	46	15	0.326086957
STOMAGEN	pCB1-8 x pJS107-2	3	76	36	0.473684211
STOMAGEN	pCB1-8 x pJS107-2	6	52	21	0.403846154
STOMAGEN	pCB1-8 x pJS107-2	3.9	106	30	0.283018868
STOMAGEN	pCB1-8 x pJS107-2	3.14	139	29	0.208633094

Development • Supplementary information

Table S2. List of plasmids used in this study

Plasmid ID	Description	Insert	Vector	BacR	PlantR	Note	Ref
pTK106	pCRII-EPF1	EPF1 cDNA	pCRII	Kan	NA	to make pTK109	This study
pTK107	pCRII-EPF2	EPF2 cDNA	pCRII	Kan	NA	to make pTK110	This study
pTK109	pBnUASPTn-EPF1	EPF1 cDNA	pBnUASPTn	Amp	NA	to make pTK112	This study
pTK110	pBnUASPTn-EPF2	EPF2 cDNA	pBnUASPTn	Amp	NA	to make pTK113	This study
pTK112	HSCRE-EPF1	EPF1 cDNA	pGII227-HSCREN2	Kan	Hyg	cre-lox system with EPF1	This study
pTK113	HSCRE-EPF2	EPF2 cDNA	pGII227-HSCREN2	Kan	Hyg	cre-lox system with EPF2	This study
pJS104	pCRII-STOMAGEN	STOMAGEN cDNA	pcR 2.1 TOPO	Amp Kan	NA	to make pJS105	This study
pJS105	pBnUASPTn-STOMAGEN	STOMAGEN cDNA	pBnUASPTn	Amp	NA	to make pJS107	This study
pJS107	HSCRE-STOMSGEN	STOMAGEN cDNA	pGII227-HSCREN2	Kan	Hyg	cre-lox system with STOMAGEN	This study
pBnUASPTn	UAS::MCS	NA	PB2n	Amp	NA	Intermediate plasmid to insert gene of interest under Gal4 UAS	Heidstra et al. 2004
pGII227-HSCREN2	Hspro::CRE	NA	pGII227	Kan	Hyg	HS promoter driving CRE. UAS with MCS. To make pTK112, 113	Heidstra et al. 2006
pCB1	35S::loxCRT1lox:Gal4-UAS-GFPER	NA	pGreen0229	Kan	Basta	Selectable marker CRT1 intercepted by two lox sites to separate Gal4 from 35S promoter. UAS-GFPER	Heidstra et al. 2006

Development • Supplementary information

Table S3. List of primers used in this study

Primer Name	Sequence	Purpose	Note
STOMGEN_F	TGTAGTTCAAGCCTCAAGACCTC	qRT-PCR STOMAGEN	This study
STOMSGEN_rc	ACTCGTTGTACGTACAAGTTGGT	qRT-PCR STOMAGEN	This study
Act2 5'intergenic_F	AAAGTTTGTACGGATCGACCA	qRT-PCR ACTIN2	Han et al. 2018
Act2 5'intergenic_rc	CGAATTTGAATATGAGCGATGA	qRT-PCR ACTIN2	Han et al. 2018
EPF1_qRT_F	ATGCCGTCTTGTGATGGTTAG	qRT-PCR EPF1	Qi et al. 2017
EPF1_qRT_rc	TCAAGGGACAGGGTAGGACTT	qRT-PCR EPF1	Qi et al. 2017
pGII227-HSCREN2.2380.fw	CTCTTCGCTATTACGCCAGCT	pGII227-HS-CRE-N2: check for inserts	This study
pGII227-HSCREN2.2968.rc	AGTGAGCTGATACCGCTCGCC	pGII227-HS-CRE-N2: check for inserts	This study
pCB1_35Sp_fw	CTGCAAGGCGATTAAGTTGGGTAAC	pCB1: check for 35S pro	This study
pCB1_35Sp_rc	AACCGGCAGCAGTTCGACATA	pCB1: check for 35S pro	This study
CRT1(F)	TTCTATGATATCCTCTTCCGCTGTG	pCB1: check for CRT1	This study
CRT1 (R)	GCGTCAGGAACCTGCGACCGTC	pCB1: check for CRT1	This study
pCB1_GAL4_fw	TTGCCGGTCTTGCGATGATTA	pCB1: check for Gal4	This study
pCB1_GAL4_rc	TGATTACGCCAAGCTCGGAATTAAC	pCB1: check for Gal4	This study
pCB1_ERGFP_fw	GCGGTAATACGGTTATCCAC	pCB1: check for erGFP	This study
pCB1_ERGFP_rc	TCTCAAACAAACACATACAGCGACT	pCB1: check for erGFP	This study
pBnUASPTn (F)	TACCGGGCCCCCCCTCGAAATTCCTG	pTK112/3: not actually in T-DNA	This study
pG7HSCREseq (F)	GTTTACAATTTCGCGCCATTC	pTK112/3: check for UAS::EPF1/2	This study
EPF1_cDNA_EcoRI (R)	CCGGAATTCTCAAGGGACAGGGTAGGACTTAT	pTK112: check for UAS::EPF1	This study
CREcDNA (F)	ATGTCCAATTTACTGACCGTACACC	pTK112/3: check for CRE	This study
CREcDNA (R)	CTAATCGCCATCTTCCAGCAGGCGC	pTK112/3: check for CRE	This study
BAMHI-EPFL9F	CGCGGATCCATGAAGCATGAAATGATGAACA	Molecular cloning STOMAGEN	This study
EcoRI-EPFL9withstop.rc	CCGGAATTCTTATCTATGACAAACACATCTATAA	Molecular cloning STOMAGEN	This study
MfeI-EPFL9withstop.rc	CCGCAATTGTTATCTATGACAAACACATCTATAA	Molecular cloning STOMAGEN	This study

Development • Supplementary information
