# Supplementary material for: Effective range of non-cell autonomous activator and inhibitor peptides specifying plant stomatal patterning
Source: Development. 2020 Sep 11;147(17):dev192237. doi: 10.1242/dev.192237 (PMC7502594; doi:10.1242/dev.192237)
Supplement: Reviewer comments [file develop-147-192237_review_history.pdf]

Effective range of non-cell autonomous activator and inhibitor peptides specifying plant stomatal patterning

Scott M. Zeng, Emily K. W. Lo, Bryna J. Hazelton, Miguel F. Morales and Keiko U. Torii
DOI: 10.1242/dev.192237

Editor: Yka Helariutta

Review timeline
Original submission: 29 April 2020
Editorial decision: 8 June 2020
First revision received: 19 June 2020
Accepted: 27 July 2020

Original submission

First decision letter

MS ID#: DEVELOP/2020/192237

MS TITLE: Nature and effective range of non-cell autonomous activator and inhibitor peptides specifying plant stomatal patterning

AUTHORS: Scott Zeng, Emily K. W. Lo, Bryna J Hazelton, Miguel F Morales, and Keiko Torii

I have now received all the referees' reports on the above manuscript, and have reached a decision. The referees' comments are appended below, or you can access them online: please go to BenchPress and click on the 'Manuscripts with Decisions' queue in the Author Area.

As you will see, the referees express considerable interest in your work, but have some significant criticisms and recommend a substantial revision of your manuscript before we can consider publication. If you are able to revise the manuscript along the lines suggested, which may involve further experiments, I will be happy receive a revised version of the manuscript. Your revised paper will be re-reviewed by one or more of the original referees, and acceptance of your manuscript will depend on your addressing satisfactorily the reviewers' major concerns. Please also note that Development will normally permit only one round of major revision.

We are aware that you may currently be unable to access the lab to undertake experimental revisions. If it would be helpful, we encourage you to contact us to discuss your revision in greater detail. Please send us a point-by-point response indicating where you are able to address concerns raised (either experimentally or by changes to the text) and where you will not be able to do so within the normal timeframe of a revision. We will then provide further guidance. Please also note that we are happy to extend revision timeframes as necessary.

Please attend to all of the reviewers' comments and ensure that you clearly highlight all changes made in the revised manuscript. Please avoid using 'Tracked changes' in Word files as these are lost in PDF conversion. I should be grateful if you would also provide a point-by-point response detailing how you have dealt with the points raised by the reviewers in the 'Response to Reviewers' box. If you do not agree with any of their criticisms or suggestions please explain clearly why this is so.

© 2020. Published by The Company of Biologists under the terms of the Creative Commons Attribution License (https://creativecommons.org/licenses/by/4.0/).

Reviewer 1

Advance summary and potential significance to field

It is not too hard to demonstrate non-cell-autonomous behavior of signaling peptides in plants, but it is notoriously difficult to quantify their action range. This study did it! The main novelty of this study lies in the development of the computational pipeline SPACE, which is a brilliant repacking of an established statistical method in Astrophysics. This method will be useful to quantitatively characterize the spatial distribution of not only stomata, but also other epidermal features such as trichomes or pigment spots (presumably with some modifications). The utilization of the Cre-lox-GAL4/UAS system to generate mosaic sectors and to avoid potential interference of fluorescent protein tags is also noteworthy. In addition the writing is very clear.

Comments for the author

I have only a few minor Comments for the authors to consider.

Title: The word "nature" in the title is a little ambiguous, could be deleted.

Line 187-189. For people who are not familiar with the cell size of Arabidopsis cotyledons, it would be helpful to indicate approximately how many cells does 100 um represent: one cell two cells?

Line 316-329. Is there any plausible hypothesis explaining the longer action range of EPF1 than STOMAGEN in terms of biochemical properties of the peptides (e.g., different post-translational modifications)?

Line 383. "Reference [2]": the reference needs to be spelt out.

Reviewer 2

Advance summary and potential significance to field

Zeng et al. harness the Cre/Lox system to create leaf sectors to locally overexpress an inhibitory or a promoting peptide of stomatal development. They then use microscopy to identify the heat-shock induced sectors and quantify stomatal density and index proximal and distal to the sectors to determine the activity range and spatial propagation of these distinct peptide signals. To do so the authors use both manual counting methods and image-analysis software driven approaches to create coordinate maps of sectors, stomata and leaf outlines. They then use a remarkable approach borrowed from astrophysics to perform a spatial point analysis that is based on a probabilistic distribution of stomata as a function of distance from sectors, which is creatively termed SPACE. This method allows the authors to statistically describe the kind of effect the peptide overexpressing sectors have on stomatal patterning (promoting or inhibitory) and apparently also the effective range of the expressed peptides.

This is an elegant and innovative approach to solve some of the limitations that classical metrics like stomatal density and index have, namely looking at distribution of features from a local to global scale, which might help to further dissect the finely tuned signaling machinery that patterns stomata across leaf surfaces and integrate a plethora of intrinsic and extrinsic/environmental signals. Although complex, SPACE is well explained and visualized and the whole manuscript is well written and easy to follow. In addition and as specified by the authors, this kind of analysis could prove useful for the analysis of pattern formation in both the animal and plant kingdom and thus nicely fits the scope of this journal.

I have a few concerns, however, that I would like to see addressed.

© 2020. Published by The Company of Biologists under the terms of the Creative Commons Attribution License (https://creativecommons.org/licenses/by/4.0/).

Comments for the author

Major concerns

1. Effective Range of Peptides The authors make very bold claims regarding the effective range of peptides but do not elaborate on how they define 60um for Stomagen and 170um for EPF1 (line 271ff). I assume they use the correlation plot in Figure 5C and basically the first intersection of the y=0, which is at 60um for Stomagen but at 100um for EPF1 rather than 170um. First the authors have to explain how they define effective range in much more detail, and then also phrase their statement much more carefully. First, They use an artificial system where the peptides are driven by Gal4/UAS, which might induce transcription at very different levels than in the endogenous context. Thus concentration at point of origin is different from the endogenous context, which might create steeper and longer gradients. I would thus argue that only relative ranges of effectiveness rather than absolute ranges of effectiveness can be determined. In addition, Stomagen expressed from the L1 layer is analyzed here, which is not its natural context and therefore could influence how far the peptide can travel and/or be effective.
These aspects (artificial system, relative vs. absolute range, Stomagen in L1) need to be extensively discussed, the method how the "effective range" is determined specified, the claim of absolute effective range strongly weakened and the EPF1 range changed from 170um to 100um.

2. Quantitative analysis of effective range In Figure 3, the authors undertake the remarkable effort to digitalize the coordinates of whole cotyledons with sectors and stomata. What I don't understand is why the authors split Figure 3 and Figure S3. Particularly when looking at panel 3G and S3C having these analyses combined would make much more sense. Also, I don't understand that the authors go through the hassle of digitalizing all these images and then don't do more finely dissected sectors to experimentally determine the range of these peptides rather than with SPACE only. I would expect the authors to use cotyledons with single sectors or sectors far apart (like in Fig. 5A upper right panel) to analyze stomatal density in consecutive ranges every 50um (i.e. 0-50um 50-100um, 100-150um, 150-200um, 200-250um and 250-300um). This would in fact show experimentally at which range these peptides act in their system and would be a strong complementation of the SPACE analysis.
Finally and importantly, the authors simply ignore that leaves with EPF1 sectors show a stomatal density effect throughout the cotyledon as shown by Fig. 3F and S3B. Please elaborate on this in the discussion and state this also in the results. Throughout the text, this observation is ignored and needs to be weakened or even changed (e.g. final statement on line 275ff, discussion line 365ff).

3. Representation of manually counted data I don't appreciate the fact that all the manually counted stomatal index data is combined and plotted as bar charts without indicating the variation of data in Figure 1 and 2 and chi-square tests are run instead of variation analyzed. I assume that variation is too big to produce statistically significant differences particularly for Stomagen, but I would nonetheless request that this data is presented with box plots and single data point jitter (like in Figure 3) and reanalyzed with a variation based statistical tests. Even if the differences are not significant anymore (for Stomagen), the trend should still be clearly visible and can be explained with stomatal index measurements being very variable on small areas. In addition, the stomatal density measurement in Fig. 3 are clearly significant for both peptides and thus a non-significant value for Fig. 1 and 2 would not weaken the manuscript in the least.

4. SPACE.
In the methods the authors state that multiple sectors were aggregated for analysis to exclude repeated measurements of overlapping sectors. While I can grasp why this is probably necessary from a technical aspect, I am afraid that it might influence the actual analysis strongly and might make no sense at all in terms of biology. Please elaborate on this in the methods and argue why this is still biologically meaningful. Alternatively, I encourage the authors to exclude leaves with more than one sector from the analysis.
Furthermore, the authors filter for sectors outside of a specific range of sector areas. First of all the range is not specified and I think this is actually a missed opportunity. Rather than excluding too big or too small sectors the authors could bin sectors and actually estimate the effect of sector size. If there is an absolute effective range then it should not matter how big the sectors are, if the sector size matters a lot then effective range is indeed concentration dependent which again would

© 2020. Published by The Company of Biologists under the terms of the Creative Commons Attribution License (https://creativecommons.org/licenses/by/4.0/).

change the authors conclusions about determining absolute rather than relative effective range (see comment 1).

Minor concerns:

5. Figure 4 In panel G,H and I uniformly spaced distribution is represented rather than repulsive inhibitory distributions as indicated by the title in G and within the text (lines 236-238). This creates massive confusion and should be changed.

6. Figure references Many Figure references to Figure 1 and Figure 2 between line 131 and 177 are wrong. There are, for example, no Figs. 2E, F and G.

7. Methods:
- Generally, the methods could benefit from careful language and grammar editing as it is much less well written than the rest of the manuscript.
- Please add all the plasmids to Table S2 that were used for crosses specified in Table S1. Also many of the plasmid names are not self explanatory and it would help to add some details within the cloning section (line 392ff, eg. What is pGII277-HSCREN2?).
- Could you elaborate on why exactly EPF2 did not work. Simply because it acts earlier in the lineage is not a convincing argument.
- on line 443-444 please specify the changes you made to the images in Adobe Photoshop.
- Geometric sectors on line 463 are actually the virtual sectors in Figure 1. Please be consistent between main text, figures and methods.

8. Figures:
Figure 1:
- please change the time of heat shock in Fig. 1A. It is specified as 15min for microscopy, 1h for RT-PCR in the methods but 2h are indicated in the Figure.
- even though this system is published, I would appreciate it if the authors would mention what CRT1 is in the figure legend, since they show it in Fig. 1B.
- in Figure 1D should be Col-0 rather than Col.

Figure 2:
- please specify what kind of sector we are looking at in A (Stom-Ox, EPF1-Ox)
- Generally the figure shows a lot of white space. Maybe the inserts/zoom-ins are actually sufficient in B.

Figure 3:
- scale bar in B is illegible
- what are the read and green jitter dots in E and F? Please explain or change.
- please change the legend and show either boxplot only for area and points/jitter only for genotype.

Figure 4:
See comment 5.

Figure 5:
Please label y-axis in C.

Typos, minor changes, etc.

112: demonstrates rather than demonstrate and the title case is inconsistent.
129: heat-sock treatment, even though very funny, should be heat-shock treatment.
156: I don't understand this (incomplete?) sentence.
195: Figure S3 rather than S2.
220: "ant" instead of "and"
350ff: here the authors speak about inter tissue communication. I would strongly encourage to cite Dow et al. 2017 New Phyt in addition to Lundgren et al. 2019 that shows that this is likely a two-way process where mesophyll influences stomata but also stomatal patterning and density influence mesophyll.

© 2020. Published by The Company of Biologists under the terms of the Creative Commons Attribution License (https://creativecommons.org/licenses/by/4.0/).

366: wrong Figure reference.
823 and 850: STOMSGEN instead of STOMAGEN?

Reviewer 3

Advance summary and potential significance to field

The authors investigate plants in which sectors overexpress, after Cre-lox mediated recombination, two ligands EPF1 and EPFL9 known to operate in the stomatal differentiation pathway. EPF1 is normally expressed in stomatal precursor cells within the epidermis, and inhibits stomatal differentiation in neighbor cells. EPFL9 (stomagen), is expressed in underlying mesophyll cells competes with EPF1 and hence acts as an activator. The genetic mosaics generated by the authors nicely confirm the previously proposed roles for EPF1 and stomagen using classical stomatal density measurements and a control system for sector size, and in addition show that EPF1 can also operate from the mesophyll layer. Both ligands produce effects outside of the sectors in which they are expressed, confirming their non-cell autonomous activity. A statistical framework adopted from physics, calculating spatial correlations is then used to determine more precisely the range of action of both ligands.
The data are consistent with, and more precise than, the classical methods and convincingly indicate that EPF1 acts at a longer range than Stomagen in these experiments.

Comments for the author

The observation that the induced sectors confirm proposed roles for EPF1 and Stomagen is useful, and the spatial correlation function worked out here is a useful tool to analyze biological patterns. My main reservation resolves around the usefulness of the ranges determined here. The sectors overexpress the ligands about 6 to 8 fold, quantified on a total tissue basis (figure 1C). However, I cannot find in the methods whether this quantification was corrected for sector sizes, which would be needed to assess how high RNA levels are within the sector. On top of that, we do not know the protein levels resulting from that. In that light, it becomes hard to determine how far these ligands were expressed above physiological levels. While this may not affect the relative differences in range between the two ligands that much, it leaves open the question what the range of the native ligands is. It would already help to correct the quantifications for sector size in the RT-PCR. In this way, the authors could discuss better the relevance of their findings for the stomatal patterning network. Right now, the discussion is mostly on the statistical tool and not on the biological relevance of the data so there is a fit to the ‘techniques and resources’ section.

First revision

Author response to reviewers' comments

We thank three expert Reviewers for positive, thoughtful, and constructive critiques. We have revised our manuscript by fully incorporating all review comments.

Our response are in boldface.

Reviewer 1

It is not too hard to demonstrate non-cell-autonomous behavior of signaling peptides in plants, but it is notoriously difficult to quantify their action range. This study did it! The main novelty of this study lies in the development of the computational pipeline SPACE, which is a brilliant repacking of an established statistical method in Astrophysics. This method will be useful to quantitatively characterize the spatial distribution of not only stomata, but also other epidermal features such as trichomes or pigment spots (presumably with some modifications). The utilization of the Cre-lox-GAL4/UAS system to generate mosaic sectors and to avoid potential interference of fluorescent

© 2020. Published by The Company of Biologists under the terms of the Creative Commons Attribution License (https://creativecommons.org/licenses/by/4.0/).

protein tags is also noteworthy. In addition, the writing is very clear.

We thank Reviewer 1 for the enthusiastic and supportive comments, recognizing our SPACE analysis as "a brilliant repacking of an statistical method in Astrophysics".

I have only a few minor comments for the authors to consider.

Title: The word "nature" in the title is a little ambiguous, could be deleted.

The word "nature" meant "inherent property", but we agree that it sounds somewhat ambiguous. As suggested, we deleted it. The revised title is: "Effective range of non-cell autonomous activator and inhibitor peptides specifying plant stomatal patterning".

Line 187-189. For people who are not familiar with the cell size of Arabidopsis cotyledons, it would be helpful to indicate approximately how many cells does 100 um represent: one cell, two cells?

We appreciate Reviewer 1's question. Cells, especially pavement cells have complex geometry, so it depends on the angle and orientation. In cotyledon epidermis we used, the average one-cell spacing is 40.9 ± 15.65 um, average size of stomata is 22.30 ± 4.29 um (longer side) and 15.32 ± 2.22 um (shorter side), and average size of non-stomatal pavement cells is 87.11 ± 30.92 um (longer side) and 19.97 ± 6.73 um (shorter side). Therefore, on average, an effective range of 100 um corresponds to 2.45 cells of spacing, 1.15-5.0 pavement cells, and 4.48-6.52 stomata. With these numbers, we could estimate that the effective range of EPF1 and Stomagen goes beyond one cell, clearly supporting the diffusive nature of these peptides. In the revision, we describe the cell size data in the Result section (lines 198-204) and in Discussion (lines 363-369).

Line 316-329. Is there any plausible hypothesis explaining the longer action range of EPF1 than STOMAGEN in terms of biochemical properties of the peptides (e.g., different post- translational modifications)?

We thank Reviewer 1 for raising a very interesting point. Although the NMR structure of Stomagen has been resolved and that of EPF1 modeled (Ohki et al. 2011 Nature Commun), nothing is known about the actual diffusion capacity of EPF/EPFL-family peptide. Likewise, both EPF2 and Stomagen exhibit similar binding affinity to corresponding receptors, ERf and TMM (Lee et al. 2015 Nature; Lin et al. 2017 Genes Dev). Interestingly, the loop domain of EPF1 and Stomagen are structurally distinct, with EPF1 having an additional disulfide bond within the loop domain, which might be providing stability (Ohki et al. 2011). Importantly, domain-swap analysis has shown that these loop domain determines specificity of Stomagen and EPF2 as an activator and inhibitor of stomatal development (Ohki et al. 2011). Whereas, such a structural difference might contribute to the difference in the action range of EPF1 vs. Stomagen peptide, currently there is no experimental evidence to support or refute such a hypothesis.

We would like to respectfully emphasize that, as Reviewer 1 recognizes" it is notoriously difficult to quantify their action range", the innovation of our approach is to quantitatively determine the effective range of peptide signals, rather than determining their actual diffusion/movement. The effective range of peptides is not solely determined by the physical and biochemical properties of the peptides, but also by signal transduction and cellular response within the context of epidermal development. Having said that, we truly value the insightful suggestion from Reviewer 1. In our revised manuscript, we discuss our finding in the context of peptide biochemical properties (lines 351-360).

Line 383. "Reference [2]": the reference needs to be spelt out.

Thank you for catching the error. It is now spelled out (Heidstra et al. 2004).

© 2020. Published by The Company of Biologists under the terms of the Creative Commons Attribution License (https://creativecommons.org/licenses/by/4.0/).

Reviewer 2

Zeng et al. harness the Cre/Lox system to create leaf sectors to locally overexpress an inhibitory or a promoting peptide of stomatal development. They then use microscopy to identify the heat-shock induced sectors and quantify stomatal density and index proximal and distal to the sectors to determine the activity range and spatial propagation of these distinct peptide signals. To do so the authors use both manual counting methods and image-analysis software driven approaches to create coordinate maps of sectors, stomata and leaf outlines. They then use a remarkable approach borrowed from astrophysics to perform a spatial point analysis that is based on a probabilistic distribution of stomata as a function of distance from sectors, which is creatively termed SPACE. This method allows the authors to statistically describe the kind of effect the peptide overexpressing sectors have on stomatal patterning (promoting or inhibitory) and apparently also the effective range of the expressed peptides.

This is an elegant and innovative approach to solve some of the limitations that classical metrics like stomatal density and index have, namely looking at distribution of features from a local to global scale, which might help to further dissect the finely tuned signaling machinery that patterns stomata across leaf surfaces and integrate a plethora of intrinsic and extrinsic/environmental signals. Although complex, SPACE is well explained and visualized and the whole manuscript is well written and easy to follow. In addition and as specified by the authors, this kind of analysis could prove useful for the analysis of pattern formation in both the animal and plant kingdom and thus nicely fits the scope of this journal.

We truly thank Reviewer 2 for recognizing our work as "an elegant and innovative approach to solve some of the limitations" in classic stomatal biology, and finding our SPACE analysis applicable to broader plant and animal kingdoms.

I have a few concerns, however, that I would like to see addressed. Major concerns
1. Effective Range of Peptides
The authors make very bold claims regarding the effective range of peptides but do not elaborate on how they define 60um for Stomagen and 170um for EPF1 (line 271ff). I assume they use the correlation plot in Figure 5C and basically the first intersection of the y=0, which is at 60um for Stomagen but at 100um for EPF1 rather than 170um. First the authors have to explain how they define effective range in much more detail, and then also phrase their statement much more carefully. First, They use an artificial system where the peptides are driven by Gal4/UAS, which might induce transcription at very different levels than in the endogenous context. Thus concentration at point of origin is different from the endogenous context, which might create steeper and longer gradients. I would thus argue that only relative ranges of effectiveness rather than absolute ranges of effectiveness can be determined. In addition, Stomagen expressed from the L1 layer is analyzed here, which is not its natural context and therefore could influence how far the peptide can travel and/or be effective. These aspects (artificial system, relative vs. absolute range, Stomagen in L1) need to be extensively discussed, the method how the "effective range" is determined specified, the claim of absolute effective range strongly weakened and the EPF1 range changed from 170um to 100um.

We thank Reviewer 2 for raising important points. As both Reviewers 2 and 3 commented, we are harnessing the Cre-Lox Gal4/UAS-based recombination system to achieve overexpression of EPF1 and Stomagen within small sectors in cotyledons. While we fully concur with Reviewer 2 that these peptides are not expressed in their endogenous 'natural' context, we would like to reiterate that the purpose of our work is to compare and quantify the effective action range of the two peptides of opposite functions during epidermal development. To address this question, we investigated <u>how far can EPF1 and Stomagen influence stomatal patterning if they are expressed at equivalent levels in the similarly confined areas, i.e. mosaic sectors</u>. Characterizing their endogenous expression levels in endogenous expression domains will not address this question, because such an analysis will reveal the 'wild-type' stomatal patterning phenotype. In a strict sense, use of any genetic perturbation, such as a cell-type specific knockdown or use of mutant backgrounds, will abrogate the endogenous contexts due to perturbation of regulatory circuits and a complex web of feedbacks. Likewise, fusing visual tags (e.g. fluorescent protein) to small peptides could alter their diffusion or activity, and will not strictly address their effective range even if they were expressed under the control of

© 2020. Published by The Company of Biologists under the terms of the Creative Commons Attribution License (https://creativecommons.org/licenses/by/4.0/).

their endogenous promoters.

Having said that, we agree with Reviewer 2 that a caveat of the mosaic overexpression approach should be discussed in the manuscript. Please see the Introduction (lines 99-101) and Discussion (lines 308-315). in our revised manuscript.

We also thank Reviewer 2 for the valid point regarding the absolute vs. relative effective range. The spatial autocorrelation statistics can quantitatively determine the probabilistic distribution of stomatal patterning in a function of distance from sector boundaries, which we define as the effective range. Thus, whereas these are not relative assumptions, we agree with Reviewer 2 that our claim might have been too strong. We revised our manuscript to soften our argument throughout the manuscript.

2. Quantitative analysis of effective range

In Figure 3, the authors undertake the remarkable effort to digitalize the coordinates of whole cotyledons with sectors and stomata. What I don't understand is why the authors split Figure 3 and Figure S3. Particularly when looking at panel 3G and S3C having these analyses combined would make much more sense. Also, I don't understand that the authors go through the hassle of digitalizing all these images and then don't do more finely dissected sectors to experimentally determine the range of these peptides rather than with SPACE only. I would expect the authors to use cotyledons with single sectors or sectors far apart (like in Fig. 5A upper right panel) to analyze stomatal density in consecutive ranges every 50um (i.e. 0-50um, 50-100um, 100- 150um, 150-200um, 200-250um and 250-300um). This would in fact show experimentally at which range these peptides act in their system and would be a strong complementation of the SPACE analysis.

As a Developmental Biologist, the corresponding author (Torii) wholeheartedly appreciates this particular comment raised by Reviewer 2. In fact, when we initiated this project some seven years ago, we took <u>the exact same approach</u> as suggested by Reviewer 2 - make consecutive bin ranges in every fixed distance (i.e. 0-50um, 50-100um, 100-150um, 150-200um, 200-250um and 250-300um, and so on). Shortly after, this approach ultimately demonstrated failure, which motivated Torii to seek input from Astrophysicists and develop the SPACE computational pipeline. Here are the reasons why.

Most importantly, binning in a fixed distance (e.g. 100 um and 200 um intervals as shown in Fig. 3 and Fig. S3, respectively) will provide some rough idea of the peptides' effective ranges, but the resolutions will not be high enough to compare the effective range of different peptides and draw conclusions. <u>In order to obtain the precise effective range, one has to narrow the bin range-- in theory, to an infinitesimally small size. However, because a given cell has its finite size, it will be impossible to quantify, or even assign stomata vs. non-stomatal pavement cells in a statistically meaningful way if we try to estimate the precise effective range</u>. This concept is analogous to integrals. Let's assume that we determine the area of a function (f(x)dx = some complicated function). We can estimate the area by a sum of rectangle areas separated by consecutive bin ranges. To accurately determine the actual area, however, the bin range must be infinitesimally small, i.e. taking an integral of the function. Not only do we face a conundrum of more precise binning leading to less precise estimation of effective range, but the consecutive bin range approach is also highly labile to the geometry, size, and location of each sector as well as the geometry of the cotyledon harboring the sector (see diagram below). In our manuscript, we exclude those areas when the discretized bin range exceed the cotyledon boundary, as absolutely zero stomata that exist outside of the cotyledon (see Method). This arbitrarily influences the area of each bin, making the analysis statistically labile.

As far as we understand, the SPACE pipeline we developed here is highly robust to geometry, size, and location of a given sector. This is because we register ALL stomata on the given cotyledon and systematically calculate the shortest distance of every single stoma from the sector boundary. This will be compared with the shortest distance of all randomly-placed points (=randomly placed virtual stomata) that are identical in number to the experimental observation. This comparison is reiterated 1000 times to give robustness to the system. The effective range can be quantified at the distance where the probabilistic distribution of stomata becomes statistically indistinguishable from that of the control sectors (see diagram

© 2020. Published by The Company of Biologists under the terms of the Creative Commons Attribution License (https://creativecommons.org/licenses/by/4.0/).

below).

In conclusion, we hope that we can convince the Reviewers that the precise binning approach is not a strong complementation of the SPACE pipeline. In the revised manuscript, we did our best to rephrase our sentence to make these points clear.

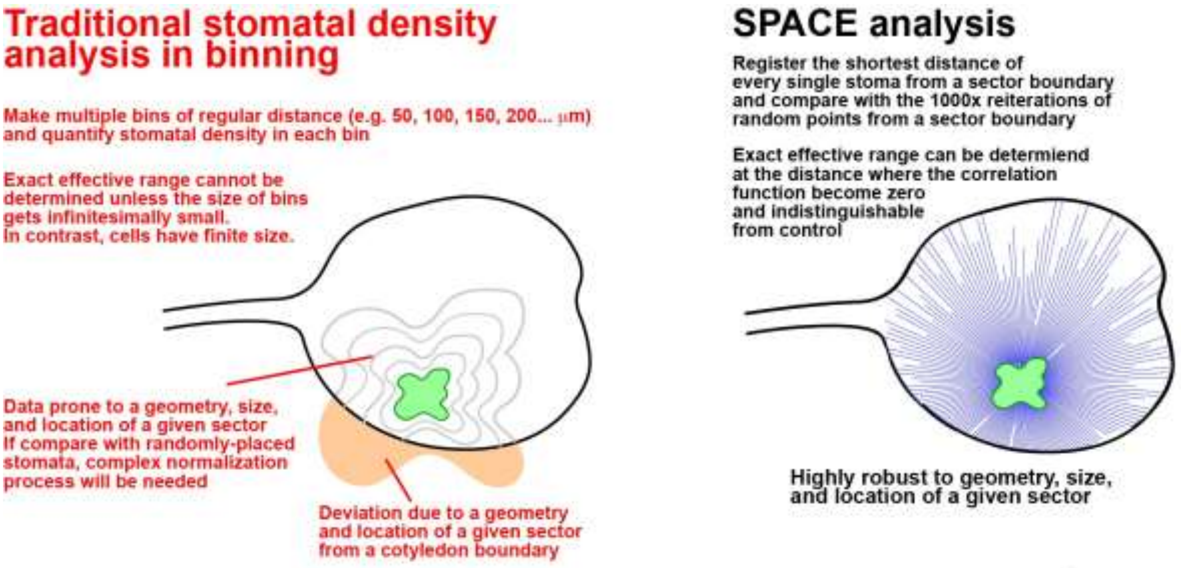

Finally and importantly, the authors simply ignore that leaves with EPF1 sectors show a stomatal density effect throughout the cotyledon as shown by Fig. 3F and S3B. Please elaborate on this in the discussion and state this also in the results. Throughout the text, this observation is ignored and needs to be weakened or even changed (e.g. final statement on line 275ff, discussion line 365ff).

We sincerely thank Reviewer 2 for commenting on the effects of EPF1 sectors on stomatal density. Although stomatal density beyond the range of interest (ROI; i.e. beyond 100 um in Fig. 3F and 200 um in Fig. S3B) in EPF1-ox sectors appears lower, this is due the way we combined the region beyond the ROI. So, it is not that we ignored the observation. To clarify, <u>we performed a consecutive bin range analysis to the stomatal density data, as suggested by Reviewer 2 in Major Point 2 (see Figure below)</u>. As one can see, at 400 um, no statistical difference (by t-test) can be detected between control vs. EPF1-ox sectors, indicating that <u>the effects of EPF1-ox sectors decay by the 400 um range</u>. In the revised manuscript, we have commented on this (e.g. lines 222-226) and replaced the original Fig. S3C, which was simply replotting of Fig. S3A and B. Thank you very much for the keen observation.

© 2020. Published by The Company of Biologists under the terms of the Creative Commons Attribution License (https://creativecommons.org/licenses/by/4.0/).

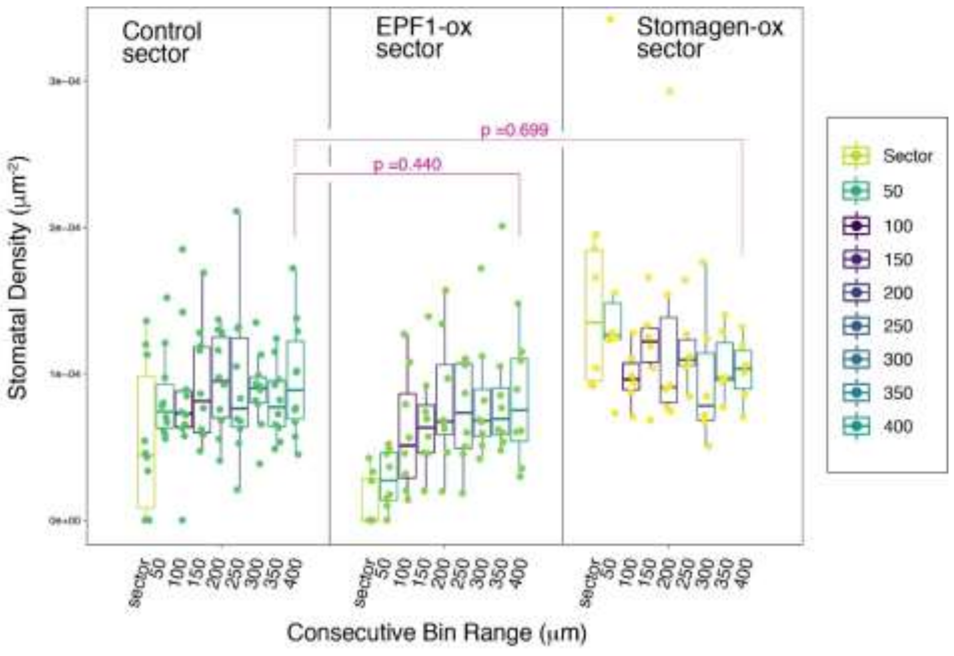

3. Representation of manually counted data
I don't appreciate the fact that all the manually counted stomatal index data is combined and plotted as bar charts without indicating the variation of data in Figure 1 and 2 and chi-square tests are run instead of variation analyzed. I assume that variation is too big to produce statistically significant differences particularly for Stomagen, but I would nonetheless request that this data is presented with box plots and single data point jitter (like in Figure 3) and reanalyzed with a variation based statistical tests. Even if the differences are not significant anymore (for Stomagen), the trend should still be clearly visible and can be explained with stomatal index measurements being very variable on small areas. In addition, the stomatal density measurement in Fig. 3 are clearly significant for both peptides and thus a non- significant value for Fig. 1 and 2 would not weaken the manuscript in the least.

As Reviewer 2 correctly points out, the purpose of aggregating all data and plotting bar graphs is to present overall effects with statistical robustness. Because we aggregated all data, chi-square analysis was done (t-test or ANOVA cannot be used for this type of dataset). As suggested by Reviewer 2, we re-analyzed stomatal index from individual sectors for both Fig. 1 and Fig. 2, presented box plots with individual data points overlaid as jitter, and performed statistical test (Welch's two sample t-test, comparing to control sector data). While the variations are large, the statistical test still shows significance in all EPF1-ox sector data against that of control sectors, as well as the statistical significance of Stomagen-ox sectors except for the 1 cell away sectors. By contrast, the virtual geometric sector data are statistically indistinguishable from control sectors. The boxplots are now provided as Fig. S1D-F and Fig. S2D-E and described in the main text (lines 164-167; lines 182-184).

4. SPACE.
In the methods the authors state that multiple sectors were aggregated for analysis to exclude repeated measurements of overlapping sectors. While I can grasp why this is probably necessary from a technical aspect, I am afraid that it might influence the actual analysis strongly and might make no sense at all in terms of biology. Please elaborate on this in the methods and argue why this is still biologically meaningful. Alternatively, I encourage the authors to exclude leaves with more than one sector from the analysis. Furthermore, the authors filter for sectors outside of a specific range of sector areas. First of all the range is not specified and I think this is actually a missed opportunity. Rather than excluding too big or too small sectors the authors could bin sectors and actually estimate the effect of sector size. If there is an absolute effective range then it should not matter how big the sectors are, if the sector size matters a lot then effective range is indeed concentration dependent which again would change the authors conclusions about determining absolute rather than relative effective range (see comment 1).

© 2020. Published by The Company of Biologists under the terms of the Creative Commons Attribution License (https://creativecommons.org/licenses/by/4.0/).

We appreciate the critique of Reviewer 2. Similar to our response to Comment #2, because each sector is highly variable in size, geometry, and location, aggregating data would provide the robust probabilistic distribution of peptide effective range that is not able to be addressed otherwise. We respectfully argue that with 95 % confidence interval, our SPACE analysis provides quantitative information, for the first time, of the properties and effective range of activator and inhibitor peptides controlling 2-D spatial patterning. As for the biological meaningfulness of aggregated data, if the reviewer's argument is extended to other common analyses, such as RNA-seq, ChIP-seq, proteome analyses, etc, that they would also be biologically meaningless unless single cells are analyzed without any data aggregation. While in some cases such analyses are possible thanks to technological and computational advancements, the individual variations could be too high to draw conclusions.

At the same time, we sincerely thank Reviewer 2 for the excellent and keen suggestion about the sector size filtering. We performed SPACE analysis with or without sector size filtering and calculated correlation functions. As provided in the Fig. S4, filtering sector size or removing the size limit did not make any essential difference in the effective ranges of EPF1 and Stomagen peptides. Accordingly, we revised the Results (lines 292-298) as well as the Methods (lines 576-578). Thank you so much for the insightful suggestion.

Minor concerns:

5. Figure 4
In panel G,H and I uniformly spaced distribution is represented rather than repulsive inhibitory distributions as indicated by the title in G and within the text (lines 236-238). This creates massive confusion and should be changed.

We thank Reviewer 2 for catching this. We have changed Figure 4G, H, I to "Uniformly spaced distribution".

6. Figure references
Many Figure references to Figure 1 and Figure 2 between line 131 and 177 are wrong. There are, for example, no Figs. 2E, F and G.

We sincerely apologize for the labeling errors. Fixed.

7. Methods:
- Generally, the methods could benefit from careful language and grammar editing as it is much less well written than the rest of the manuscript.

Thank you for highlighting this section for us. As requested, we did grammar editing throughout the Method section.

- Please add all the plasmids to Table S2 that were used for crosses specified in Table S1. Also many of the plasmid names are not self explanatory and it would help to add some details within the cloning section (line 392ff, eg. What is pGII277-HSCREN2?).To be done

These plasmids were generated by Dr. Renze Heidstra (Wageningen Univ., the Netherlands). In response to Reviewer 2's request, we now include these published plasmids in Table S2 and refer to the Heidstra et al. 2004 paper. In addition, we provided a brief description of the Heidstra plasmids in the Method Section ("Molecular Cloning and Generation of Transgenic Plants").

Specific explanations added:
"pBnUASPTn, a transient vector to clone cDNA of interest under Gal4 UAS" " pGII277-HSCREN2 vector, which contains heat-shock promoter driven CRE recombinase gene" "pCB1, which carries 35S::lox-CRT1-lox: Gal4-UAS-GFPER (Heidstra et al., 2004)"

- Could you elaborate on why exactly EPF2 did not work. Simply because it acts earlier in the lineage is not a convincing argument.

© 2020. Published by The Company of Biologists under the terms of the Creative Commons Attribution License (https://creativecommons.org/licenses/by/4.0/).

We thank Reviewer 2 for raising an important point. It is well established that EPF2 acts on the initial stomatal lineage entry (Hunt and Gray 2009 Curr Biol; Hara et al. 2009 PCP; Lee et al. 2012 Genes Dev). In our detailed time-lapse analysis of stomatal cell-state transition during germinating cotyledon development (e.g. Peterson et al. 2013 JoVE; Han et al. 2018 Dev Cell), the initial commitment to stomata, as monitored by the onset of MUTE protein accumulation, occurs ~30 hours after germination. After extensive optimization, we determined that the heat shock treatment 24 hours after germination is the best condition to reliable generate reproducible size and number of sectors. This means, while the CreLox recombination is occurring to produce EPF2-overexpressing sectors, the initial EPF2-regulated stomatal lineage transition has already passed. As a result, we do not see statistically-significant inhibition of stomatal development by EPF2- ox sector experiments.

On the other hand, EPF1 acts at the later stage of stomatal differentiation and enforces one-cell spacing of stomata (Hara et al. 2007), and it has been shown the EPF1-ox or matureEPF1 peptide application inhibits MUTE protein accumulation, leading to arrested meristemoids (Lee et al. 2012 Genes Dev, Qi et al. 2017 eLife). Stomagen acts antagonistic to EPF2 (Lee et al. 2015 Nature), but also to EPF1 (Qi et al. 2019 BioRxiv).

Review of available literature, our time-lapse analysis of detailed stomatal-lineage marker expression during germination, and our heat-shock CreLox experiments emphasize the strict developmental time window of EPF2 action, which is too early to address due to our technical limitation. While it is out-of-scope of this manuscript, in the future, it would be interesting to apply the heat-shock-induced recombination to a whole plant (or region around the shoot meristem) to test the role of EPF2-ox sectors in emerging rosette leaf primordia. We revised our Method section to make the argument more convincing and less confusing to readers (lines 473-485). Thank you very much for the suggestion.

- on line 443-444 please specify the changes you made to the images in Adobe Photoshop.

Added as requested.

- Geometric sectors on line 463 are actually the virtual sectors in Figure 1. Please be consistent between main text, figures and methods.

Geometric sectors are virtual sectors because they are superimpositions of GFP mosaic sector geometries to wild-type epidermis. While writing the original manuscript, we discussed how to name them, and decided to use "geometric sectors" to avoid misinterpretation due to a colloquial use of a word 'virtual' in the modern era (e.g. virtual reality). We clarified this throughout the manuscript to be consistent. Thank you for pointing this out.

8. Figures: Figure 1:
- please change the time of heat shock in Fig. 1A. It is specified as 15min for microscopy, 1h for RT-PCR in the methods but 2h are indicated in the Figure.

Thanks for catching the typo. Fixed.

- even though this system is published, I would appreciate it if the authors would mention what CRT1 is in the figure legend, since they show it in Fig. 1B.

As suggested, we added extensive description of the published CreLox system by Heidstra et al. into the Fig. 1B legend.

"(top) heat-shock promoter drives expression of CRE recombinase (light blue), which acts on lox recombination sites (black triangles) to cleave off the *E.uredovora CRT1* gene (yellow); (bottom) removal of insulator gene *CRT1* now drives *Gal4* (peach) under the control of CaMV35S promoter. The Gal4 protein binds to the UAS at the promoter of *erGFP* (green) as well as *EPF* peptide gene (purple). The resulting erGFP protein will mark the mosaic sectors, whereas EPF peptides will be secreted to apoplast."

© 2020. Published by The Company of Biologists under the terms of the Creative Commons Attribution License (https://creativecommons.org/licenses/by/4.0/).

- in Figure 1D should be Col-0 rather than Col.

Corrected.

Figure 2:
- please specify what kind of sector we are looking at in A (Stom-Ox, EPF1-Ox)

It is a control sector. We now indicate in the figure panel and legend.

- Generally the figure shows a lot of white space. Maybe the inserts/zoom-ins are actually sufficient in B.

As suggested, we inserted Zoom-in images as insets Panel B.

Figure 3:
- scale bar in B is illegible

Fixed.

- what are the read and green jitter dots in E and F? Please explain or change.

Black jitter dots represent individual data points. The red dot in E and the green dot in F are outliers (Q3 + 1.5*IQR). We matched the color of outliers to the respective boxplot.
We revised the figure legend to make this clear. Thank you.

- please change the legend and show either boxplot only for area and points/jitter only for genotype.

Changed as suggested.

Figure 4:
See comment 5.

Again, we thank Reviewer 2 for catching this. We have changed Figure 4G, H, I to "Uniformly-spaced distribution".

Figure 5:
Please label y-axis in C.

Labeled as "Spatial Correlation Function (Probability from Random Distribution) ".

Typos, minor changes, etc.

112: demonstrates rather than demonstrate and the title case is inconsistent.

Corrected.

129: heat-sock treatment, even though very funny, should be heat-shock treatment.

Corrected. Thanks for catching this. It was funny, indeed...

156: I don't understand this (incomplete?) sentence.

Corrected. The complete sentence should read as below. We apologize for the oversight (lines 157-160).

Changed to: "On the other hand, stomatal index in cells adjacent (1 cell away) to control

© 2020. Published by The Company of Biologists under the terms of the Creative Commons Attribution License (https://creativecommons.org/licenses/by/4.0/).

empty sectors or neighboring the adjacent cells (2 cells away) was not statistically different from either control empty sectors or heat-shocked wild-type geometric sectors (Fig. 1F-G)".

195: Figure S3 rather than S2.

Corrected. Thank you.

220: "ant" instead of "and"

Corrected. Thank you...

350ff: here the authors speak about inter tissue communication. I would strongly encourage to cite Dow et al. 2017 New Phyt in addition to Lundgren et al. 2019 that shows that this is likely a two-way process where mesophyll influences stomata but also stomatal patterning and density influence mesophyll.

This is an excellent suggestion. As Reviewer 2 emphasizes, the Dow et al. 2017 manuscript revealed the roles of EPFs, Stomagen, ERf, and TMM on mesophyll development. We now incorporate their findings in our Discussion (lines 397-401). We thank Reviewer 2 for insightful advice.

366: wrong Figure reference.

Corrected to (Figs. 3D-G, 5C). Thank you for catching this.

823 and 850: STOMSGEN instead of STOMAGEN?

We apologize for the typo. Corrected.

Reviewer 3

The authors investigate plants in which sectors overexpress, after Cre-lox mediated recombination, two ligands EPF1 and EPFL9 known to operate in the stomatal differentiation pathway. EPF1 is normally expressed in stomatal precursor cells within the epidermis, and inhibits stomatal differentiation in neighbor cells. EPFL9 (stomagen), is expressed in underlying mesophyll cells, competes with EPF1 and hence acts as an activator. The genetic mosaics generated by the authors nicely confirm the previously proposed roles for EPF1 and stomagen using classical stomatal density measurements and a control system for sector size, and in addition show that EPF1 can also operate from the mesophyll layer. Both ligands produce effects outside of the sectors in which they are expressed, confirming their non-cell autonomous activity. A statistical framework adopted from physics, calculating spatial correlations, is then used to determine more precisely the range of action of both ligands. The data are consistent with, and more precise than, the classical methods and convincingly indicate that EPF1 acts at a longer range than Stomagen in these experiments.

We thank Reviewer 3 for accurately summarizing the key discovery of our work and finding our pipeline "a useful tool to analyze biological patterns".

The observation that the induced sectors confirm proposed roles for EPF1 and Stomagen is useful, and the spatial correlation function worked out here is a useful tool to analyze biological patterns. My main reservation resolves around the usefulness of the ranges determined here. The sectors overexpress the ligands about 6 to 8 fold, quantified on a total tissue basis (figure 1C). However, I cannot find in the methods whether this quantification was corrected for sector sizes, which would be needed to assess how high RNA levels are within the sector. On top of that, we do not know the protein levels resulting from that. In that light, it becomes hard to determine how far these ligands were expressed above physiological levels. While this may not affect the relative differences in range between the two ligands that much, it leaves open the question what the range of the native ligands is. It would already help to correct the quantifications for sector size in the RT-PCR. In this way, the authors could discuss better the relevance of their findings for the

© 2020. Published by The Company of Biologists under the terms of the Creative Commons Attribution License (https://creativecommons.org/licenses/by/4.0/).

stomatal patterning network. Right now, the discussion is mostly on the statistical tool and not on the biological relevance of the data so there is a fit to the 'techniques and resources' section.

We sincerely thank Reviewer 3 for the insightful comments. First, we would like to clarify that the qRT-PCR analysis was performed to test that the Heat-shock induced CreLox Gal4/USA system indeed works to overexpress *EPF1* and S*TOMAGEN*. This is a starting point of our project. To do so, 24 hour-post germination seedlings were subjected to a heat-shock treatment for 1 hour. Under this extended heat-shock condition, nearly the entire cotyledon achieves recombination, and we can detect strong upregulation of *EPF1* and *STOMAGEN* transcripts. On the other hand, the seedlings were heat-shocked for 15 min to generate one (or two) small mosaic sectors in the cotyledon epidermis for analyzing effective range. Detecting the level of expression fold change in these small sectors by qRT-PCR (after grinding seedlings) is extremely difficult. There was a typo in our original schematic diagram of the experimental design (Fig. 1A). It should have read 15 min (for generating mosaics). We sincerely apologize for the oversight, which may have made a confusion.

As Reviewer 3 (and also Reviewer 2) points out, our system confers mosaic sector overexpression above physiological levels. We would like to respectfully reiterate that we investigated how far can EPF1 and Stomagen influence stomatal patterning if they are expressed at equivalent levels in the similarly confined areas, i.e. mosaic sectors. This way, one can compare the efficacy and action range of these two peptides.
Characterizing their endogenous expression levels in endogenous expression domains will not address this question, because such an analysis will reveal 'wild-type' stomatal patterning phenotype. In a strict sense, use of any genetic perturbation, such as cell-type specific knockdown or use of mutant backgrounds, will abrogate the endogenous contexts due to perturbation of regulatory circuits and a complex web of feedbacks.
Likewise, fusing visual tags (e.g. fluorescent protein) to small peptides would alter their diffusion or activity, and will not strictly address their effective range even if they were expressed under the control of their endogenous promoters.

We fully acknowledge the Reviewer 3's concern that multiple factors influence the effective range, including the actual RNA levels of peptide genes as well as translation, processing, and secretion of mature peptides. During the revision process, we tried to see whether normalization of spatial autocorrelation with sector size would yield meaningful results.

As we described in the Methods, spatial autocorrelation can be determined by:

$$Correlation(r_i) = \frac{S(r_i)}{< R(r_i) >} - 1$$

Where S(ri) is the number of stomata counted between a distance of rI and ri+1 away from a sector. <R(ri>)> is the expected value of the number of random points counted between a distance of rI and ri+1 away from a sector, estimated by averaging the number of points counted in that range of distance for 1000 random distributions.

We tried to normalize by individual sector size, then the correlation function would look like:

$$\frac{w_1 S_1 + w_2 S_2 + w_3 S_3 + w_4 S_4 + ... + w_5 S_n}{w_1 < R_1 > + w_2 < R_2 > + w_3 < R_3 > + w_4 < R_4 > + ... + w_5 < R_n >}$$

Where S1 is the number of stomata counted between a distance of rI and ri+1 away from a sector of a given sector that are normalized by size function □1. R1 is the expected value of the number of random points counted between a distance of rI and ri+1 away from a sector, estimated by averaging the number of points counted in that range of distance for 1000 random distributions, normalized by size function □1.

This will be mathematically too complicated. Moreover, we have to make unrealistic assumptions on linear relationships among RNA concentration, peptide concentration within the cell, mature peptide concentration outside of the cell, and their concentration in the effective range. We would like to reiterate that by using the SPACE pipeline, we are able to

© 2020. Published by The Company of Biologists under the terms of the Creative Commons Attribution License (https://creativecommons.org/licenses/by/4.0/).

quantitatively characterize the effective peptide ranges by plotting the correlation function (a probabilistic distribution of stomatal patterns in the function of distance). At the same time, we acknowledge that we have been too strong on the 'absolute' vs 'relative' effective range.

We truly thank Reviewer 3 for encouraging us to discuss the possible biological implications of our finding. Well-known mathematical framework has revealed a critical role of short-range activator and long-range inhibitor for two-dimensional patterns (Turing patterning). Whereas Stomagen does not directly induce EPF1, indirectly, Stomagen will lead to generation of cell types (stomatal precursors) that express EPF1. Thus, the difference in their effective range may imply some universal theme on the activator-inhibitor relationship in biological patterning. In addition, the effective range of Stomagen roughly corresponds to 3 stomata (see our response to Reviewer 1). This might imply that non-cell autonomous effects of Stomagen can be easily buffered by the robust EPF1/2-peptide signaling. In the revised manuscript, we softened our conclusion regarding the precise (absolute) effective range, and further elaborated the biological relevance of our discovery (e.g. see: lines 99-101; 270-274; 308-315; 363-369).

Second decision letter

MS ID#: DEVELOP/2020/192237

MS TITLE: Effective range of non-cell autonomous activator and inhibitor peptides specifying plant stomatal patterning

AUTHORS: Scott M. Zeng, Emily K. W. Lo, Bryna J. Hazelton, Miguel F. Morales, and Keiko U. Torii
ARTICLE TYPE: Research Article

I am happy to tell you that your manuscript has been accepted for publication in Development, pending our standard ethics checks.

Reviewer 1

Advance summary and potential significance to field

Already summarized in the initial review, and the reviewers seemed unanimous about the significance of the paper.

Comments for the author

The authors have done a fantastic job in the revision. Definitely addressed all of my comments and as far as I can tell, also fully addressed other reviewers'
comments.

Reviewer 2

Advance summary and potential significance to field

The authors addressed all of my comments thoroughly and respectfully. I particularly appreciated their patience in carefully laying out the advantage versus limitation of SPACE vs. manual binning to determine range of effectiveness.

Comments for the author

I have two small suggestion but I leave it up to the authors and the editor whether they want to heed them:

© 2020. Published by The Company of Biologists under the terms of the Creative Commons Attribution License (https://creativecommons.org/licenses/by/4.0/).

1. First, the SPACE vs. manual binning plot the authors provided in their response to my major concern 2 really helped me understand the limitation of manual binning and would in my opinion make a very helpful supplementary figure.
2. Second, I really appreciate the authors making the effort to include manually binned data in Fig. S3C. While it clearly shows that the highest ranges are not statistically different anymore, I would appreciate statistical comparisons on all the distances between controls and overexpression sectors.

That being said, I would like to thank the authors again for a respectful and thorough response to my concerns.

Reviewer 3

Advance summary and potential significance to field

The authors have adequately responded to my questions. They have further corroborated their analysis by direct comparisons to other methods. Most importantly, they have further clarified the usefulness of their new metric for stomatal biology: it serves as a range test that is sufficient to support an indirect Turing mechanism for stomatal density control.

Comments for the author

In my view, the manuscript is ready for publication.

© 2020. Published by The Company of Biologists under the terms of the Creative Commons Attribution License (https://creativecommons.org/licenses/by/4.0/).
